# Genes and pathways underlying regional and cell type changes in Alzheimer's disease

**DOI:** 10.1186/gm452

**Published:** 2013-05-25

**Authors:** Jeremy A Miller, Randall L Woltjer, Jeff M Goodenbour, Steve Horvath, Daniel H Geschwind

**Affiliations:** 1 Interdepartmental Program for Neuroscience and Human Genetics Department, UCLA, 2309 Gonda Bldg, 695 Charles E Young Dr. South, Los Angeles, CA 90095-1761, USA; 2 Department of Pathology, Oregon Health & Science University, Department of Pathology L113, Portland, OR 97239, USA; 3 Human Genetics Department, UCLA, 2309 Gonda Bldg, 695 Charles E Young Dr. South, Los Angeles, CA 90095-1761, USA; 4 Human Genetics Department and Biostatistics Department, UCLA, 4357A Gonda Bldg, 695 Charles E Young Dr. South, Los Angeles, CA 90095-1761, USA; 5 Human Genetics Department and Neurology Department, UCLA, 2309 Gonda Bldg, 695 Charles E Young Dr. South, Los Angeles, CA 90095-1761, USA

## Abstract

**Background:**

Transcriptional studies suggest Alzheimer's disease (AD) involves dysfunction of many cellular pathways, including synaptic transmission, cytoskeletal dynamics, energetics, and apoptosis. Despite known progression of AD pathologies, it is unclear how such striking regional vulnerability occurs, or which genes play causative roles in disease progression.

**Methods:**

To address these issues, we performed a large-scale transcriptional analysis in the CA1 and relatively less vulnerable CA3 brain regions of individuals with advanced AD and nondemented controls. In our study, we assessed differential gene expression across region and disease status, compared our results to previous studies of similar design, and performed an unbiased co-expression analysis using weighted gene co-expression network analysis (WGCNA). Several disease genes were identified and validated using qRT-PCR.

**Results:**

We find disease signatures consistent with several previous microarray studies, then extend these results to show a relationship between disease status and brain region. Specifically, genes showing decreased expression with AD progression tend to show enrichment in CA3 (and vice versa), suggesting transcription levels may reflect a region's vulnerability to disease. Additionally, we find several candidate vulnerability (ABCA1, MT1H, PDK4, RHOBTB3) and protection (FAM13A1, LINGO2, UNC13C) genes based on expression patterns. Finally, we use a systems-biology approach based on WGCNA to uncover disease-relevant expression patterns for major cell types, including pathways consistent with a key role for early microglial activation in AD.

**Conclusions:**

These results paint a picture of AD as a multifaceted disease involving slight transcriptional changes in many genes between regions, coupled with a systemic immune response, gliosis, and neurodegeneration. Despite this complexity, we find that a consistent picture of gene expression in AD is emerging.

## Background

Alzheimer's disease (AD) is the most common form of dementia, affecting nearly half of the population over the age of 85 years [1]. AD has no cure and although <10% of cases can be linked to genetic mutations in *PSEN1*, *PSEN2*, or *APP*, the majority of AD cases have no known genetic cause, and the underlying genetic modifiers are highly complex and remain elusive [2]. While neurofibrillary tangles (NFTs) and amyloid deposition are pathological hallmarks of AD, transcriptional studies suggest that dysfunction of cellular pathways such as energy metabolism [3-5], synaptic transmission [3-6], and myelin-axon interactions [3] may precede the neuropathological indicators [7,8]. Other pathways implicated in AD include inflammation [3,4,9], cytoskeletal dynamics [9,10], signal transduction [3,4,9,11], protein misfolding [3,12], transcription factors [3,9], and cell proliferation [3,9]. Furthermore, these transcriptional changes do not occur throughout the brain in a uniform manner; AD follows a well-characterized progression, with pathology beginning in brain areas involved in learning, memory, perception, and emotion, such as the entorhinal cortex, amygdala, and hippocampus, then spreading throughout the cortex [7,13]. This regional vulnerability is strikingly apparent in the hippocampus, where CA1 pyramidal neurons are invariably affected earlier and more severely than their neighboring CA3 counterparts.

While many of these transcriptional changes are likely due to dysfunctional cellular pathways, changes in the cellular composition of affected brain regions are also likely to impact gene expression levels [14]. In addition to widespread pyramidal cell loss and diffuse atrophy of affected brain regions [13], the role of glial cells in AD pathophysiology is becoming more apparent. Microglia, the resident immune cells in the central nervous system, have been shown to cluster around amyloid plaques [15], increasing in number in the early stages of AD [16]. Reactive astrocytes show similar response to disease pathology, whereas astrocytes not associated with pathology tend to degenerate [17]. Oligodendrocyte dysfunction has also been suggested as an early event in AD progression [18]. Although a few groups have used methods such as laser capture microdissection [19,20] and microaspiration [6] to enrich their samples for transcripts expressed in pyramidal neurons, the extent to which cellular composition impacts gene expression remains unclear.

To address these issues and to complement these forward genetic analyses, we have performed a large-scale transcriptional analysis in brain of individuals with advanced AD and non-demented controls, focusing specifically on the CA1 field of the hippocampus and the relatively less affected adjacent region, CA3. For comparisons between brain regions and across disease status, we find consistency between our results and several previous studies; however, with the addition of CA3 samples in AD we are also able to provide novel insights into AD pathophysiology. In CA1 we find that genes related to synaptic transmission and cell-cell signaling tend to show decreased expression in AD, whereas genes related to cell death and cell proliferation tend to show increased expression. Interestingly, many of the changes occurring in CA1 also occur in CA3, although to a lesser extent. Furthermore, genes showing decreased expression with AD progression are likely to also show an initial enrichment in CA3, whereas genes showing increased expression with AD progression are likely to also show an initial enrichment in CA1, indicating that transcription levels in a region may reflect that region's vulnerability to disease. Based on this rubric, we identify *ABCA1, MT1H, PDK4*, and *RHOBTB3 *as putative vulnerability genes and *FAM13A1, LINGO2*, and *UNC13C *as putative protection genes. To account for the changes in cellular composition that occur in AD, we developed and apply a linear model, finding that the most differentially expressed genes are likely involved in dysfunctional cellular pathways rather than due to cell loss or gliosis. Along the same lines, we use weighted gene co-expression network analysis (WGCNA) to find modules of highly co-expressed genes enriched with markers for major cell types, each of which shows a distinct expression pattern that provides insight into aging and AD. Of particular note is a microglia-associated module that shows increased expression in controls with early signs of NFT pathology, lending support to the idea that microglial activation may be one of the earliest events in AD progression. Together, these findings suggest that large-scale regional vulnerabilities in AD are likely due to the combination of many small differences in gene expression patterns between brain regions, affecting multiple cell types.

## Materials and methods

### Tissue collection

De-identified, pathological specimens consisting of fresh-frozen human hippocampus and frontal cortex samples were generously provided by two tissue centers (Alzheimer's Disease Center, Oregon Health and Sciences University, and Human Brain and Spinal Fluid Resource Center), both from clinically and neuropathologically classified late-onset AD-affected individuals, as well as from age- and sex-matched controls (Table 1; Additional file 1). The research was performed at UCLA, but because the study does not use data or specimens from living individuals, it was not deemed by the UCLA Institutional Review Board as subject to review.

**Table 1:** Summary of subject information

Category	Control	AD	*P*-value
Gender	11 M/5 F	9 M/8 F	0.37
Age	81.7 ± 6.9	77.3 ± 9.1	0.13
PMI	10.8 ± 6.8	11.2 ± 6.3	0.85
Plaques	0.58 ± 0.51	2.59 ± 0.51	6.0E-11
Braak	1.50 ± 0.52	5.33 ± 0.62	6.0E-14

Control and AD groups are controlled for gender, age at death (years), and PMI (postmortem interval in hours) as closely as possible, but there is a significant difference in plaque burden (0 to 3; 0 = none, 1 = sparse, 2 = moderate, and 3 = severe) and Braak stage (0 to 6) [13] between groups, as expected. Values are mean ± standard deviation. Gender indicates the number of subjects of each gender (M, male; F, female) per group.

Subjects from the Alzheimer's Disease Center fell into two categories. First, control subjects were participants in brain aging studies at the Oregon Aging/Alzheimer's Disease Center. Subjects received annual neurological and neuropsychological evaluation, with clinical dementia rating assigned by an experienced clinician. Controls had normal cognitive and functional examinations. Second, the AD subjects were diagnosed by a clinical team consensus conference, met National Institute for Neurological and Communicative Disorders and Stroke-Alzheimer's Disease and Related Disorder Association diagnostic criteria for clinical AD, had a clinical dementia rating of greater than 1.0, and neuropathologic confirmation at autopsy (after informed consent). Tissue use conformed to institutional review board-approved protocols. Subjects from the Spinal Fluid Resource Center met comparable criteria. Ordinal scales were used to assess NFT burden (Braak stage of 0 to 6) [13] and amyloid plaque burden (0 to 3), where higher scores indicate greater pathology.

### Tissue processing, RNA isolation, and expression profiling

Hippocampal CA1 and CA3 subfields were isolated using the following method. First, frozen tissue was cut into 60 μm sections, with the first section from each sample stained with cresyl violet. Labeled sections were then photographed and enlarged, and these images were used as reference during dissections. Using a scalpel CA1 and CA3 subfields were dissected from sectioned but unlabeled tissue on dry ice and immediately placed into elution buffer for RNA extraction. Total RNA from each sample was isolated using the RNeasy Micro Kit with DNase I treatment (QIAGEN, Valencia, CA, USA), then tested for quality on the Agilent 2100 Bioanalyzer using RNA 6000 Nano Chips (Agilent Technologies, Palo Alto, CA, USA). For each of the 71 samples passing RNA quality control standards, 360 ng total RNA were sent to the Southern California Genotyping Consortium (Los Angeles, CA, USA) for analysis on the Illumina HumanHT-12 v3 Expression BeadChips (Illumina, San Diego, CA, USA). Samples were randomly assigned to BeadChips in order to minimize the impact of any batch effects on differential expression by region or disease status.

### Microarray analysis

Unprocessed expression data for all 71 samples have been deposited in NCBI's Gene Expression Omnibus (GEO) [21] and are accessible through GEO Series accession number GSE29378. Illumina HumanHT-12 v3 Expression BeadChips measure the expression of over 25,000 annotated genes using 48,803 probes. Initial expression values were computed from probe intensities using the program GenomeStudio (Illumina). From these data, six samples with low inter-array correlation were removed as outliers (as described in [5]). The data were then quantile normalized. Two final outlier arrays were removed as above, for a total of 63 samples (32 control, 31 AD) remaining in the analysis. This outlier removal procedure is completely unbiased, since it ignores phenotypic traits.

After preprocessing and outlier removal, the following categories of probes were omitted from the analysis: (i) probes called as present (*P *< 0.1) in three or fewer samples; (ii) probes not assigned gene symbol annotations; and (iii) duplicate probes for a single gene, but only if these probes had a Pearson's correlation value of R > 0.8 (using the function collapseRows [22]). When removing duplicate probes for a gene, the probe with the highest average expression level was retained. This final filtering step left a total of 23,696 probes in our analysis corresponding to 17,128 genes. The resulting expression matrix is also available from the same location.

### Differential expression analysis

We measured differential expression with respect to region, disease, and Braak stage, often using only a subset of the total data. Unless otherwise specified, an uncorrected *P*-value cutoff of <0.05 (using a Student's *t*-test) combined with a fold change (FC) >1.2 was used to determine differential expression (after correcting for multiple comparisons, very few genes showed significant differential expression). When it came to validating findings across data sets, we kept track of the directionality of gene expression (for example, genes that are over-expressed in diseased individuals in one data set should also be over-expressed in the diseased individuals of another data set). For region-enrichment comparisons, paired *t*-tests were used, since CA1 and CA3 were obtained from each subject.

To characterize lists of differentially expressed genes based on gene ontology annotation, we used Enrichment Analysis Systematic Explorer (EASE) [23], as previously described [3,5]. EASE assigns identified genes to Gene Ontology (GO), Kyoto Encyclopedia of Genes and Genomes (KEGG), and other experimentally derived gene categories, and then tests for significant overrepresentation of identified genes within each category using a modified Fisher's exact test. In order to compare our differential expression results with similarly designed previous studies, we first sorted and ranked all genes in our analysis with respect to region in control only, as well as with respect to disease status in CA1 alone. We sorted and ranked the variables using the Z scores. Since a monotonically increasing function relates Z scores to *P*-values, this is equivalent to sorting by *P*-values. For each previous study, we then noted where the reported differentially expressed genes were located in our sorted list, and assessed the resulting significance using a Z score to measure divergence from a random distribution. Specifically, we quantify consistency using 'mean gene rank', which is the mean ranked differential expression of a subset of genes, scaled by the number of total genes and offset by 0.5 to set chance = 0.

We also determined putative vulnerability and protection genes with AD. Vulnerability genes are defined as genes showing significantly higher expression in CA1 than CA3 (FC >1.2) and increasing with AD to a significantly greater degree in CA1 compared with CA3 (FC in CA1 >1.2 and FC in CA1 > FC in CA3). Protection genes were defined as genes showing significantly higher expression in CA3 than CA1 (FC >1.2) and also increasing to a greater degree (FC in CA3 >1.2, FC in CA1 <1.2) or decreasing to a lesser degree (FC in CA3 <1.4 × FC in CA1) in CA3 compared with CA1. Both vulnerability and protection genes also must have a Bayes ANOVA significance of *P *< 0.05 as assessed using the function bayesAnova (parameters: conf = 12, bayes = 1, winSize = 11) [24], and all of the FC criteria must hold when defining groups based on both the mean and the median expression for each group.

To ensure that our results for region and disease status were not solely a product of neurodegeneration and gliosis, we used a multivariate linear model to regress individual gene expression levels against region, disease status, and marker genes for four major cell types: neurons (*SYT1*), astrocytes (*AQP4*), oligodendrocytes (*MOG*), and microglia (*TYROBP*), respectively. These particular marker genes met the following three criteria: 1) they had multiple publications linking them to their matched cell type; 2) they showed significant experimental confirmation in two previous microarray studies; and 3) they showed high connectivity with their matched cell type in two previous WGCNA studies in brain [14,25]. We also note that the model is fairly robust to choice of marker genes for cell type.

### Weighted gene co-expression network analysis and module characterization

We created a network from normalized expression data by following the standard procedure of WGCNA [26]. Briefly, we calculated pair-wise Pearson correlations between each gene pair, and then transformed this matrix into a signed adjacency matrix using a power function. The components of this matrix (connection strengths) were then used to calculate 'topological overlap' (TO), a robust and biologically meaningful measurement of gene similarity based on two genes' co-expression relationships with all other genes in the network. Genes were hierarchically clustered using '1 - TO' as the distance measure, and initial module assignments were determined by using a dynamic tree-cutting algorithm [27]. For computational reasons, initial module formation was performed only on the approximately 15,000 genes with the highest overall connectivity, as previously described [14]. We calculated Pearson correlations between each gene and each module eigengene - referred to as a gene's module membership - along with the corresponding *P*-values [14,28]. The module eigengene is commonly used as a representative value for a module, and is defined as the first principal component of a module, and is the component that explains the maximum possible variability for all genes in a module. For the final module characterizations, each gene was (re)assigned to the module for which it had the highest module membership. Thus, genes were each assigned to exactly one module, including genes that were omitted from the initial module formation.

Modules were characterized using the following strategy: first, modules were annotated using EASE (as described above); second, modules were further annotated by measuring their overlap with modules from previous WGCNA studies of human and mouse brain [14,25]; third, cell type annotations were confirmed by measuring the overlap between our modules and experimentally derived lists of cell type-specific genes using the function userListEnrichment [22]; fourth, modules were annotated for region and disease specificity by measuring their overlap with lists of differentially expressed genes from the six studies discussed in the text [3,4,20,29-31]; and finally, module eigengenes were associated with all phenotypic traits available in this study (region, disease, age, and so on) in order to gain insight into the role each module might play in AD pathophysiology. To test for significant overlap between gene lists from our study and those from previous lists, the hypergeometric distribution (Fisher's exact test) was used. Modules were graphically depicted using VisANT [32], as previously described [5]. Network depictions show the 250 strongest reciprocal within-module gene-gene interactions (connections) as measured by TO. A gene was considered a 'hub' if it had at least 15 depicted connections.

### Quantitative RT-PCR validations

RNA for quantitative RT-PCR (qRT-PCR) validations of eight disease- and region-specific genes was collected as for the arrays. Although RNA was collected from the same samples as in the microarray analysis, it was collected from different sections. Total RNA was collected from larger pieces of hippocampus and frontal cortex of five select individuals for qRT-PCR validations of microglial genes. For these samples, the RNeasy Mini Kit with DNase I treatment (QIAGEN) was used for RNA isolation. A list of primer pairs used for qRT-PCR validation is provided (Table S7 in Additional file 6). In total, 13 genes were assessed using qRT-PCR. For qRT-PCR validations of *PDPR*, results from two separate primer pairs were averaged.

### *In situ *hybridization validation

Probes for RNA *in situ *hybridization analysis were designed using distal forward and reverse primer pairs from two proximal qRT-PCR validation regions to yield a probe of approximately 500 bp that was cloned into the pCR4-TOPO vector (Invitrogen). To produce digitonin-labeled probes, plasmids were first linearized with NotI (New England Biolabs, Ipswich, MA, USA), then transcribed using the DIG RNA Labeling kit (Roche, Indianapolis, IN, USA) according to the manufacturer's protocols. Formalin-fixed paraffin-embedded tissue sections of control and AD case individuals cut to 16 μm thickness were obtained from the UCLA Alzheimer's Disease Research Center. Hybridization was performed according to [33] with modifications from [34] using 600 μg RNA per section.

## Results

To address the issue of regional vulnerability with disease progression, while also taking into account the complexity of AD, we performed a large genome-wide comparison of CA1 and CA3 gene expression in the brain of individuals with advanced AD and non-demented controls using Illumina Human HT-12 microarrays. The purpose of this study design was several-fold: first, to identify genes that show an association with vulnerable regions in AD progression; second, to quantify the relationship between region and disease using gene expression; third, to bring together the results of several previous studies of disparate design coming to apparently inconsistent results; fourth, to determine how the composition of cell types in hippocampus changes with AD progression; fifth, to identify genes marking early, presymptomatic signs of AD progression; and finally, to provide a gene expression resource for interested scientists. The data discussed in this publication have been deposited in NCBI's GEO [21] and are accessible through GEO Series accession number GSE29378.

To minimize the possibility of group bias, brain samples from individuals with moderate to severe AD (disease group; N = 17) were matched for gender, age, and post mortem interval (PMI) with individuals showing little to no cognitive deficits (control group; N = 16), as closely as possible (Table 1; Additional file 1). Furthermore, samples were randomly assigned to microarrays to limit batch effects. Simple clustering of the arrays reveals no significant confounding factors: samples cluster by individual, but not by batch, brain bank, location on the array, PMI, gender, or age (Figure S1 in Additional file 6). With the exception of heat shock proteins, no GO categories showed significant enrichment for genes differentially expressed with batch, brain bank, location on the array, or PMI, further suggesting that our results are properly controlled for possible confounding factors.

### Genes differentially expressed with disease or region

We first determined which genes showed differential expression with disease progression ('disease-altered' genes) in CA1 and CA3 separately, and then annotated these gene lists using EASE [23]. In CA1, we find that genes related to synaptic transmission and cell-cell signaling tend to show decreased expression with AD, whereas genes related to cell death and cell proliferation tend to show increased expression (Table 2; for a complete list of differentially expressed genes, see Additional file 2). EASE also identified two specific pathways showing increased expression with AD progression - the MAPKKK cascade and the transforming growth factor-ß signaling pathway. Both have previously been implicated in AD progression [35,36]. Similar changes are seen in CA3; however, they are less dramatic (Figure S2A in Additional file 6), which is consistent with the lesser vulnerability (relative protection) of this region to AD-related neurodegeneration compared with CA1.

**Table 2:** Annotation for lists of differentially expressed genes

Gene category	EASE score
**Down with AD (in CA1)**	
Synaptic transmission	6.18E-14
Cell-cell signaling	7.88E-12
CNS-specific functions	2.99E-05
Potassium channel activity	6.46E-05
Neurogenesis	1.35E-04
cAMP-mediated signaling	5.99E-04
Lipoprotein	1.30E-03
**Up with AD (in CA1)**	
Response to stress	2.36E-05
Cell-matrix adhesion	8.79E-04
MAPKKK cascade	2.73E-03
Polymorphism	2.95E-03
Hs_TGF beta signaling pathway	5.25E-03
Cell proliferation	1.14E-02
Cell death	1.22E-02
**Enriched in CA3 (in control)**	
Transport	2.16E-11
Neurogenesis	1.85E-08
CNS-specific functions	7.37E-08
Synaptic transmission	7.27E-07
Cell growth and/or maintenance	1.28E-05
Cytoskeletal protein binding	2.80E-04
Potassium transport	3.04E-04
Cholesterol biosynthesis	4.70E-04
**Enriched in CA1 (in control)**	
Signal transducer activity	3.42E-09
Response to external stimulus	3.70E-07
Metallothionein	9.74E-06
Immune response	1.53E-05
Cell-cell signaling	1.89E-05
Cell motility	4.31E-05
Homeostasis	9.97E-05
Polymorphism	9.48E-03

Significantly overrepresented gene ontology categories (EASE score <0.01) are presented for region- and disease-related lists of differentially expressed genes. Numerous other similar significant categories are not included to reduce redundancy. Complete lists of differentially expressed genes are presented in Additional file 2. CNS, central nervous system; TGF, transforming growth factor.

We next identified genes enriched in either CA1 or CA3 ('region-enriched' genes) in controls. Since both regions were collected from identical tissue sections, removing a major source of variability, we identified more differentially expressed genes than in the disease-related analysis. We find that the list of genes enriched in CA3 shows overrepresentation for genes involved in synaptic transmission, cytoskeletal protein binding, and cholesterol biosynthesis (Table 2; Additional file 2). In the case of CA1-enriched genes, we find over-representation of genes related to signal transduction, immune response, and cell motility (Table 2; Additional file 2). Interestingly, we also find enrichment in metallothioneins, a group of heavy metal binding proteins that have previously been implicated in aging and AD [37]. When we perform the identical region-enrichment analysis in the AD group, we see similar results as with controls; however, fewer genes meet significance (Figure S2B in Additional file 6). This attenuation of region-enriched genes with disease is consistent with previous results in ischemia [30], and is not due to increased variance in the AD samples, as the standard deviations of the genes differentially expressed in controls are no different than in AD.

To determine which genes showed the most significant gene expression changes, we further refined our lists of disease-altered and region-enriched genes, by first including only genes with a fold change >1.4, then sorting each list by *P*-value (the top ten genes of each comparison are presented in Table 3). Many of these genes are already known to have a role in AD. For example, high levels of α1-antichymotrypsin (the protein product of SERPINA3) in blood plasma have been associated with increased risk for dementia [38]. Likewise, S100A6 was found to show increased expression in both white matter as well as the subset of astrocytes that surround amyloid plaques in both humans and two transgenic mouse models of AD, suggesting that this gene may play a role in AD neuropathology [39].

**Table 3:** Top genes differentially expressed by disease and region

Gene	Fold change	*P*-value
**Genes regionally DE in control only**		
CA1-specific		
*** SPARCL1***	**-1.44 (-1.42)**	**7.0E-08**
* CYP1B1*	-1.42	4.7E-07
* PPP1R16B*	-1.49	4.2E-06
* KCNH3*	-2.07	5.2E-06
* EPHB1*	-1.47	5.8E-06
* STOX1*	-1.56	5.8E-06
* MT1M*	-1.58	1.1E-05
* ID2*	-1.49	1.4E-05
* SOX2*	-1.49	1.7E-05
* GPAM*	-1.53	1.9E-05
CA3-specific		
*** NRIP3***	**2.14 (2.52)**	**7.4E-08**
*** ABHD12***	**1.54 (1.60)**	**9.0E-07**
* TMEM158*	1.72	2.0E-06
*** TSPAN18***	**4.00 (4.07)**	**2.9E-06**
* TOMM34*	1.85	4.2E-06
* CCDC109A*	1.52	6.7E-06
* HOMER2*	1.57	6.8E-06
* CPNE4*	3.18	7.5E-06
* LINGO1*	1.65	7.8E-06
* HMGCR*	1.65	8.8E-06
**Genes changing with AD in CA1 only**		
Down with AD		
* SEPT5*	-1.59	2.8E-05
* CSPG5*	-1.82	6.9E-05
* WFDC1*	-1.47	8.2E-05
* KCNIP1*	-1.77	1.0E-04
*** CXCL14***	**-1.96 (-2.09)**	**1.1E-04**
* ANKRD20A1*	-1.81	1.6E-04
*** SEC14L5***	**-1.43 (-1.25)**	**3.0E-04**
* LOC648639*	-1.71	3.1E-04
* ARPP-21*	-1.92	3.9E-04
* ADRA1B*	-2.11	6.3E-04
Up with AD		
*** S100A6***	**1.59 (1.83)**	**4.6E-07**
*** GEM***	**1.64 (2.05)**	**5.9E-06**
* PFKFB3*	1.44	4.5E-05
* SERPINA3*	2.11	6.7E-05
* TPST1*	1.63	1.5E-04
* SPARC*	1.71	1.6E-04
* CAB39L*	1.46	1.9E-04
* RCN1*	1.42	2.0E-04
* DMN*	1.73	2.9E-04
* BCL2*	1.45	3.1E-04

Top ten CA1-specific genes in control (top list), CA3-specific genes in control (second list), genes down with AD in CA1 (third list), and genes up with AD in CA1 (bottom list) with fold change >1.4. For each list, the left column is the gene, the second column is the fold change of differential expression, and the right column is the associated *P*-value as measured by a *t*-test (Materials and methods). For genes that were validated using qRT-PCR (in bold), fold changes from the validations are presented in parentheses in the 'Fold change' column. All genes are still significant after accounting for cell type composition using a linear model. DE, differentially expressed.

Finally, we confirmed the direction and FC of eight of these highly disease-altered or region-enriched genes by qRT-PCR (Materials and methods; genes in bold in Table 3), thus validating a cross-section of our microarray results by an independent method.

### *In silico *validation shows concordance among microarray studies of Alzheimer's disease

**Figure 1:**
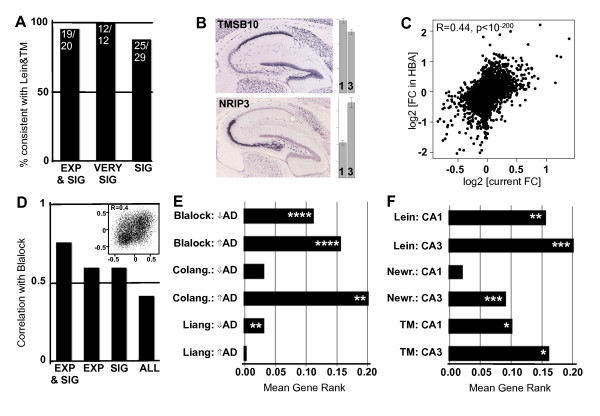
**Differential expression results are consistent with previous studies**. **(a) **Genes showing enrichment for CA1 or CA3 in previous studies of AD show similar enrichment in this study (Lein *et al. *[29], Torres-Muñoz *et al*. (TM) [31]). The y-axis shows the percentage of genes with consistent results between studies. The x-axis shows the subset of genes used: EXP, 'highly expressed' (average expression >1,000); SIG, 'significant' differential expression (*P *< 0.05); VERY SIG, 'highly significant' differential expression (*P *< 0.005). **(b) **Example mouse *in situ *hybridizations for common CA1- and CA3-enriched genes were reproduced from the Allen Mouse Brain Atlas (Allen Institute for Brain Science, ©2009 [67]). Bars represent the relative expression levels in CA1 and CA3 in our data. Error bars show standard error. **(c) **Common region-enriched genes in this study and in the Allen Human Brain Atlas [68]. Points correspond to the ratio of the average CA3 versus CA1 expression in both studies on a log2 scale. See also Figure S3 in Additional file 6. **(d) **Genes show similar correlations to disease (as measured by Braak stage) between this study and a previous study of similar design [3]. The y-axis shows the correlation of this measure across genes between studies. The x-axis labeled as in (a) (ALL, all genes). Data corresponding to all genes is presented in the inset and Figure S4 in Additional file 6. **(e) **Genes showing significant disease alteration in CA1 in three previous studies tend also to change in the same direction with AD in this study (Blalock *et al. *[3], Colangelo *et al*. (Colang.) [4], Liang *et al. *[20]). Bars represent the level of consistency between our results and the labeled list of differentially expressed genes (y-axis). Mean gene rank (x-axis) scales from 0.5 (completely opposite results) to 0.5 (perfectly consistent) with chance = 0 (see Materials and methods). *P*-values: *P < 0.05, ** *P *< 0.006, *** *P *< 0.00001, **** *P *< 10^-45^. **(f) **Genes showing significant region-enrichment in control in three previous studies tend to show similar regional enrichment in this study. Labeling as in (e) (Newrzella *et al*. (Newr.) [30]).

One of the major issues with microarray analyses, both in general and with AD specifically, is the apparent lack of agreement between studies of similar design on which genes are differentially expressed, which has introduced confusion and ambiguity in the field. To address this issue, we assessed how consistent our results were compared with previous studies finding either region-specific genes in control or disease-altered genes in CA1, by measuring how many such genes changed in the direction predicted by our results. We first compared our regional results to two previous studies of hippocampus - one in mouse [29] and one in human [31]. When we include only genes in our study with either high expression (average expression >1,000) or high levels of differential expression (*P *< 0.005), thus improving separation of the signal from the noise, we find nearly perfect agreement between our study and both previous studies (Figure 1a). Even when we lower our threshold for what we consider differentially expressed genes (*P *< 0.05) we find a very high level of agreement (86%). Specific examples of between-study agreement are presented in Figure 1b. As an added control, we compared our results with results from a recent microarray atlas of human brain gene expression [40], finding a high correlation of CA3/CA1 fold changes (R = 0.44, *P *~ 0), along with several common region-enriched genes in both studies (Figure 1c; Figure S3 in Additional file 6; Additional file 3). Likewise, when we compare our disease results to a previous study of CA1 in AD run using a similar design [3], we find high agreement, in particular when including only highly expressed and significantly differentially expressed genes (Figure 1d; Figure S4 in Additional file 6).

We next extended these analyses to all genes, including those with much more marginal differential expression, in a total of six studies: three assessing changes with AD progression in CA1 (Figure 1e) [3,4,20] and three finding CA1- and CA3-enriched genes in control hippocampus (Figure 1f) [29-31]. We ranked all of our genes from the most CA1-enriched to the most CA3-enriched (or the ones most decreasing with AD to the ones most increasing), and then compared lists of differentially expressed genes from previous studies to our ranked lists (Materials and methods). For 9 of the 12 comparisons, we find the distribution of genes significantly shifted in the expected direction of overlap, and in the three other comparisons the direction of change was still correct, but did not reach significance (Figure 1e,f). In other words, genes presented as CA3-enriched in earlier studies are significantly more likely to have higher expression in CA3 than in CA1 in this study, and likewise for the other phenotypes. Thus, despite the many differences in experimental designs between studies, this *in silico *validation indicates that there is significant and previously unappreciated concordance between functional genomic studies related to AD. These analyses highlight for the first time many common genes and pathways in AD pathogenesis, showing a degree of convergence that has not been well appreciated previously.

### Interaction between region and disease identifies factors associated with selective vulnerability

**Figure 2:**
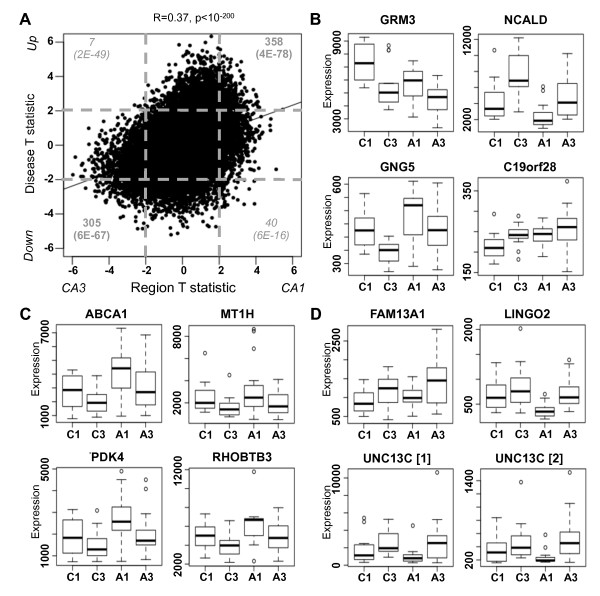
**Gene-by-region study design provides novel insights into AD**. **(a) **Region (x-axis) and disease (y-axis) T-statistics for each gene (point) are plotted, along with the line of best fit. We find a significant correlation between disease and region. The number of these genes differentially expressed with both region and disease (four corners) are displayed in grey, with *P*-values representing the significance of enrichment (in dark grey) or depletion (in italics). Dashed lines correspond to significant differential expression (*P *< 0.05). **(b) **Representative genes for each gene-by-region pattern of expression. Box plots of gene expression levels (y-axis) are displayed for each of the four groups (x-axis): CA1 in control (C1), CA3 in control (C3), CA1 in AD (A1), and CA3 in AD (A3). **(c) **The four vulnerability genes show higher expression in CA1 than CA3 and also increase with AD to a larger degree in CA1 compared with CA3. Labeling as in (b). **(d) **The three protection genes show higher expression in CA3 than CA1 and also increase with AD to a larger degree or decrease with AD to a smaller degree in CA3 compared with CA1. Note that there are two significant probes for UNC13C. Labeling as in (b).

In addition to identifying genes differentially expressed with disease and with region separately, we can also assess the interaction between disease and region. Given the highly complex and heterogeneous nature of AD, it is likely that a region's vulnerability to AD depends, in part, on the expression of large numbers of genes at slightly varying levels. To address this issue, we repeated the differential expression comparisons, this time without separating either CA1 from CA3 in our analysis of disease-altered genes, or control from AD in our analysis of region-enriched genes. We find that genes enriched in CA3 are likely to also show decreased expression with AD progression, whereas genes enriched in CA1 are also likely to show increased expression with AD progression (Figure 2a). For example, while *NCALD *shows decreased expression with AD in both brain regions, the expression levels of this gene in CA3 in AD have not even dropped below its CA1 levels in control, while the converse is true for *GNG5 *(Figure 2b). Our results are consistent with the hypothesis that brain regions with relative protection from AD pathology will also tend to show a less abnormal gene expression signature at baseline. A list of all genes showing significant differential expression with both region and disease are presented in Additional file 4.

To find genes that may play a role in the relative vulnerability of CA1 or protection of CA3, we considered the relative difference in fold change with disease between these brain regions. Our nomenclature of 'protection' and 'vulnerability' genes should be interpreted with a grain of salt, since carefully designed validation studies are needed to show a causal relationship implied by the terminology. Such a strategy has previously been successfully applied in the discovery of potential disease-related genes in AD [12] and novel neuroprotective genes in frontotemporal dementia [41]. More specifically, we would expect vulnerability genes to have higher expression levels in CA1 than CA3 and also to increase expression to a greater extent in disease, whereas protective genes should show the opposite pattern. Overall, we found four candidates for putative vulnerability genes (*ABCA1*, *MT1H*, *PDK4, RHOBTB3*; Figure 2c) and three candidates for putative protection genes (*FAM13A1, LINGO2, UNC13C*; Figure 2d) meeting these criteria (Materials and methods). Two of our four vulnerability genes have been previously associated with AD: *MT1H *is a member of the family of zinc-regulating metallothionein proteins discussed earlier, while *ABCA1 *is a major cholesterol regulator that can influence amyloid plaque aggregation and clearance (reviewed in [42]). Furthermore, increasing expression of *ABCA1 *with increasing severity of AD has been measured both functionally and neuropathologically [43]. Although none of the neuroprotective genes have known roles in AD, two have been associated with neuroprotection or plasticity in other contexts: variants of *LINGO2 *have been associated with risk and age of onset in Parkinson's disease [44], while *UNC13C *is a candidate gene for critical period neuronal plasticity in visual cortex [45].

Finally, to validate expression of *UNC13C*, we performed *in situ *hybridization on tissue from three additional human hippocampi showing no, moderate, and high pathology according to Braak and Braak staging (Figure S5 in Additional file 6). Consistent with both microarray probes for this gene, expression of *UNC13C *shows increased expression in CA3 relative to CA1 in AD tissue compared with control. These results highlight the importance of including regions of different levels of vulnerability in transcriptional studies to allow for more comprehensive disease gene assessments.

### Accounting for cell type differences occurring with disease progression

One potential variable that we wished to explore was the role of cell type differences underlying differential expression changes. For example, with neurodegeneration there will be lost neurons, increases in glial cells, and a likely infiltration of inflammatory cells. To address this issue, we created a linear model measuring differential expression with region and with disease, which also takes into account four major cell types in the brain using linear regression (Materials and methods). We chose genes used extensively in the literature as markers, and that have also been labeled as hub genes in previous transcriptional studies of human brain [14,25] (although we note that choice marker gene makes very little difference in the results). As a caveat, we point out that this linear model ignores within-subject relationships and resulting *P*-values should only be interpreted as descriptive as opposed to inferential measures.

After accounting for cell type, we found that approximately 60% of differentially expressed genes are still significant (Figure S6 in Additional file 6), and that most of the same GO categories from Table 2 still show significant enrichment, albeit to a lesser extent. This result suggests that, with relatively equal contributions, differentially expressed genes in our analysis mark two distinct phenomena: first, there are differences in cell composition between regions and disease states - a result that we will discuss extensively in the context of WGCNA below - and second, many genes show significant changes in expression even after accounting for changes in cell composition. This second category likely represents the subset of differentially expressed genes marking dysfunctional cellular pathways, which we hypothesize encompasses the most significant gene expression changes, and includes all the genes from Table 3. These results suggest that standard microarray analyses of heterogeneous tissue can accurately pinpoint genes related to dysfunctional intracellular pathways for the most highly differentially expressed genes, but that more sophisticated analyses are required to address cell type composition for the majority of such genes.

### WGCNA uncovers disease-related expression changes of major cell types

**Figure 3:**
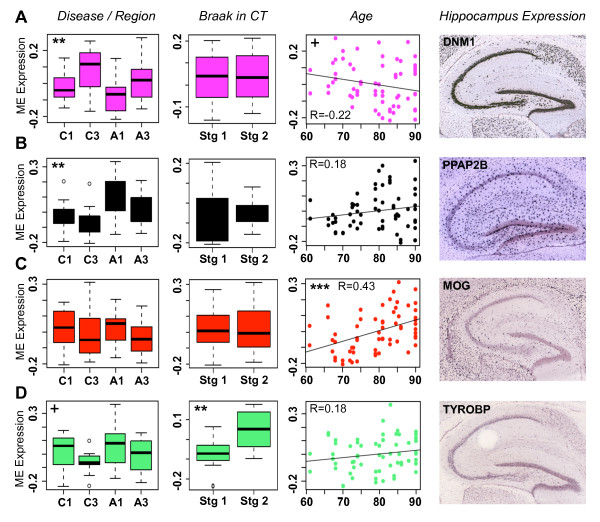
**Modules for cell type associate with disease-relevant phenotypes**. **(a-d) **Representative modules for four major cell types - pyramidal neuron (a), astrocyte (b), oligodendrocyte (c), and microglia (d) - each show significant association with a disease-relevant trait. In the first column, module association with region and disease is measured using Bayes ANOVA. Box plots are displayed for each of the four groups (x-axis, labeling as in Figure 2b). In the second column, module association with Braak stage in controls is measured using a *t*-test. Box plots are displayed for Braak stages (Stg) of 1 and 2. In the third column, Pearson correlation between module expression and age is presented, along with the line of best fit. The y-axis in all cases represents module eigengene expression. *P*-values: +, 0.05 <*P *< 0.1; **, *P *< 0.007; ***, *P *< 0.0004. In the fourth column, mouse *in situ *hybridizations for the top hub gene in each module were reproduced from the Allen Mouse Brain Atlas (Allen Institute for Brain Science, ©2009, available from [67]). These genes appear to mark the appropriate cell types, although no region-specificity is seen in any case. Note that *PPAP2B *is the top hub gene for a different astrocyte module (light cyan).

**Figure 4:**
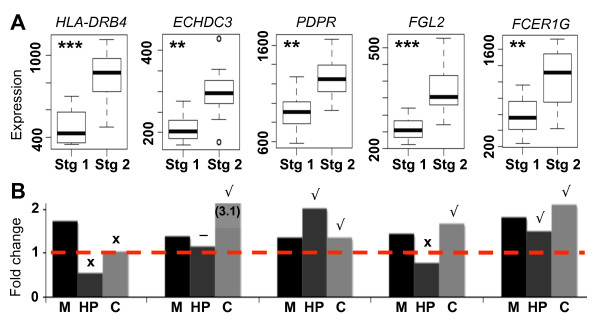
**Most genes showing increased expression with Braak stage are confirmed with qRT-PCR**. **(a) **Box plots showing expression levels (y-axis) for five of the top genes differentially expressed with Braak stage in control (x-axis). *P*-values of differential expression were measured using a *t*-test (**, *P *< 0.003; ***, *P *< 0.0004). **(b) **Fold changes (y-axis) for each of these genes between Braak stages of 1 and 2 were calculated using three methods (x-axis): microarray (M), qRT-PCR of tissue from hippocampus (HP), and qRT-PCR of tissue from frontal cortex (C). Genes were rated as confirmed (tick; fold change >1.2), marginal (minus sign; 1.1 < fold change < 1.2), and non-confirmed (cross; fold change <1.1).

To complement traditional differential expression analyses and further explore the pathophysiology of AD from a systems perspective, we performed WGCNA on our samples (Materials and methods). We found 19 modules of highly co-expressed genes (Figure S7 in Additional file 6; see Additional file 5 for more specific module information and see Figure S8 in Additional file 6 for module depictions). As with previous WGCNA studies of brain tissue [14,25,46], many of these modules correspond to cell types and to basic cellular components (Table 4). Each marker gene used in our linear model shows high connectivity in a module corresponding to that same cell type, confirming that the genes for our linear module were appropriately chosen. Furthermore, for each major cell type, we find modules associated with AD-relevant traits. For example, the module eigengenes of many neuron-associated modules show decreased expression in AD individuals compared with non-demented controls (Figure 3a). Astrocyte modules tend to have the opposite pattern, showing increased expression in AD (Figure 3b). In addition, we find one module highly enriched for oligodendrocyte markers (the red module), which does not show region or disease specificity, but is the only module positively correlated with age in controls (Figure 3c). We also find that one module marking microglia (the light green module) shows significantly increased expression in non-demented controls in Braak stage 2 compared with controls in Braak stage 1 (Figure 3d), suggesting a relationship between microglia activation and tau pathology, even in the absence of AD symptoms. Finally, as a methodological control, we evaluated the expression patterns of the top hub gene for each cell-type module using the Allen Mouse Brain Atlas resource [47]. We find that in mouse each hub gene seems to mark the correct cell type, providing further evidence that our module characterizations are valid (Figure 4).

**Table 4:** Summary of module characterization and trait association

Module	Characterization	Trait association
Black	Astrocyte	**Up with AD**
Blue	Mitochondria, neuron	*Down with AD*
Brown	**Pyramidal neuron**	Down with AD duration, *down with AD*, *enriched CA3*
Cyan	Glutatmatergic synapse, neuron	**Down with AD**, down with age, enriched in CA3
Green	Astrocyte (and other glia), cell death?	**Up with AD**
Green-yellow	Ribosome, oligodendrocyte	---
Grey60	Microglia (M8)?	*Up with AD*
Light cyan	Astrocyte	Enriched in CA1
Light green	Microglia (M10)	Enriched in CA1, up with NFTs in CT, *up with AD*
Light yellow	**Pval+ interneuron**	**Down with AD**, *enriched in CA1*
Magenta	**Pyramidal neuron**	**Down with AD, enriched CA3**
Midnight blue	Heat shock	Up with AD, down with PMI, batch, Brain Bank
Pink	Many mixed categories	---
Purple	Choroid plexus, extracellular signaling	---
Red	Oligodendrocyte, ribosome	**Up with age**
Salmon	Glia?	Up with AD
Tan	Neuron?	Down with age
Turquoise	Signal transduction	---
Yellow	Transcription, M9h	Up with AD duration, *up with AD*

For each module, summary characterizations and trait associations are presented (as described in the text). Module characterizations in bold were confirmed in [46]. Trait associations in normal font were found in this study only, those in italics were found in previous studies only, and those in bold were found in both previous studies as well as this study.

### Microglia markers are early indicators of tau pathology

To further examine the association between microglia and early tau pathology, we determined which genes showed the most significant increase in expression between Braak stages of 1 and 2 using a *t*-test, this time including CA1 and CA3 samples together to increase statistical power. Overall, we found 490 significant genes, including many in the light green 'microglial' module and >60 from the 'defense response' GO category (*P *< 10^-18^). To validate our results we performed qRT-PCR, adding two new controls to our analysis (Additional file 1). Of the five additional genes tested, three were validated (Figure 4). We then repeated the analysis on frontal cortex from the same individuals, and found that four of these genes validated (Figure 4). Since NFTs have not yet formed in CA3 or frontal cortex by Braak stage 2 and are only isolated in CA1 [13], this result suggests that microglial activation spreads throughout the brain before NFT pathology, and may therefore be one of the earliest indicators of AD progression.

**Figure 5:**
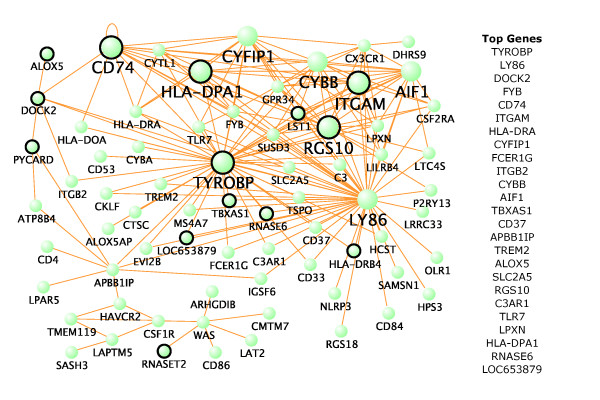
**Genes in the microglial module are related to early NFT pathology**. The top 250 gene-gene interactions of the light green module are displayed as measured by topological overlap. Larger dots represent hub genes with at least 15 connections. Circled genes are significantly up-regulated in NFT-bearing neurons (*P *< 0.03) and also overexpressed in Braak stage 2 controls (*P *< 0.03). The length of each line and the position of each node were arbitrarily chosen by VisANT to highlight network structure. The top 25 genes based on module membership are also presented.

This result does not, by itself, suggest an association between NFTs and microglia: instead it suggests that NFT pathology in the transentorhinal region and systemic microglial activation are both early presymptomatic events. To determine what, if any, association may exist between NFTs and microglia, we analyzed data from a published study of layer 2 stellate island neurons in the entorhinal cortex in subjects with mid-stage AD (GEO accession number GSE4757) [19]. In this study, laser capture microdissection was used to collect 1,000 neurons bearing NFTs and 1,000 normal neurons from the same ten subjects. From these data, we obtained a list of genes up-regulated in neurons bearing NFTs. Of the top 25 genes significantly up-regulated in NFT-bearing neurons and also overexpressed in Braak stage 2 controls (*P *< 0.03; Table S6 in Additional file 6), we find that 20 are in the light green (microglial) module, including 5 hubs (Figure 5). Together, these results suggest that microglia activation occurs early in the progression of AD and is associated with NFTs in addition to amyloid pathologies.

## Discussion

We have performed a large genome-wide analysis of gene expression in the human hippocampus in the context of AD progression. To address the issue of selective regional vulnerability - that is, why neurons die more readily and earlier in certain areas - we performed microarray-based gene expression analysis on RNA both from CA1 and the nearby, relatively less affected CA3. Using this novel study design, we find that CA3 has a less abnormal expression pattern at baseline than CA1, consistent with the observed pathological gradient in susceptibility. We also find candidate protection and vulnerability markers for AD, some of which have already been implicated in the disease (*ABCA1 *and *MT1H*). We perform an *in silico *validation of previous gene expression studies, identifying significant, previously unrecognized convergence of gene expression abnormalities in AD. Finally, we use WGCNA to find co-expression modules (which turn out to be enriched with markers for major cell types) and measure their expression in the context of aging and AD progression (Table 4). The identification of disease-associated modules permits extending the results from analysis of single genes independently, to the identification of dysregulated pathways. Of note, results from one module suggest that microglial genes show increased expression in controls of Braak stage 2.

### Inclusion of CA3 allows for an in-depth look at Alzheimer's disease pathophysiology

To the best of our knowledge, this study represents the first transcriptional snapshot of CA3 in AD human brain, although multiple transcriptional studies of dementia have compared regions of differing degrees of vulnerability in order to gain insight into disease. For example, comparisons between cortex and cerebellum in mice with and without a tau mutation led to the discovery of puromycin-sensitive aminopeptidase (PSA) as a potential tau protease acting as a neuroprotective factor in frontotemporal dementia [41]. In a microarray study of AD, Hata and colleagues [12] found that calcineurin Aß showed significantly enriched gene expression levels in hippocampus relative to parietal cortex in AD, but not in control. Using *in situ *hybridization and RT-PCR analysis, they then confirmed that this gene might play a critical role in the pathophysiological mechanisms of AD. Another study of AD in the human brain compared gene expression levels across six brain regions affected by AD at different stages of progression [20]. They found decreased expression of MAPT, CDK5, and various tubulin proteins across multiple AD-affected regions (including CA1), possibly indicating a cellular attempt to inhibit NFT formation. These studies highlight the importance of including regions with differing levels of vulnerability in the analysis of diseases in which there is a specific stereotyped progression.

There are several advantages of using CA3 as the comparison region for CA1, rather than a more distant, unaffected, region. First, CA1 and CA3 are structurally similar: they each have four layers, are directly connected via the Schaffer collateral, and are located in the hippocampus. Because of these similarities, it is more likely that differential changes with disease are due to disease pathology, rather than due to changes in local environment. Second, since both CA1 and CA3 are distinctly laminated, it is relatively easy to dissect these regions in a consistent manner between samples. Thus, we were able to collect reliable data using microscope-aided dissection. Third, the proximity of CA1 and CA3 allows us to collect samples from the same slide, thus eliminating one level of technical bias. Finally, many previous studies have compared CA1 and CA3 in control tissue, providing a valuable test of the validity of our results.

### Prominent disease-related genes

As with most microarray studies of disease, we first determined AD-related genes by finding the most differentially expressed genes between control and disease. Using this method we have found several genes, including *SERPINA3 *[38] and *S100A6 *[39], which have been previously associated with AD (Table 3). We can also find disease genes by filtering our list of differentially expressed genes using data from previous studies (for example, Figure S4E in Additional file 6). One advantage of our methodology is that we can find protection and vulnerability genes by taking into account expression levels in tissues that are differentially affected by disease progression. In this way we find *ABCA1 *[42] and *MT1H *[37], which have also been previously associated with AD (Figure 2), along with several novel disease genes. Using WGCNA we can find additional disease genes in the form of hub genes for modules correlated with AD-related traits. Previous studies have shown that hubs are more likely than other genes to be functionally relevant; for example, in the case of oncogenic signaling networks in glioblastoma, nearly all hubs of a cancer-related module were found to be molecular targets for treatment [48]. In this case we find that *RGS4*, a gene involved in calcium signaling that has been found to show decreased expression in several studies of AD [49], was the top hub gene in the light yellow module (Figure S8I in Additional file 6), which also shows decreased expression with AD (Table 4). Together, these results demonstrate that a multifaceted systems biology analysis of expression data increases a study's effectiveness in finding disease-related genes.

### Current results are consistent with previous studies of region and disease

By a number of measures, we show remarkable consistency (that was previously unrecognized) between published studies of gene expression in AD: first, genes showing increased expression with AD in CA1 are enriched for synaptic transmission and cell-cell signaling, while those decreasing with AD are enriched for cell death and proliferation genes (Table 2); second, we find that most previously published lists of genes differentially expressed by hippocampal region or disease state are consistent with our results, even if they do not on the surface appear to be in agreement with each other (Figure 1; Figures S3 and S4 in Additional file 6); and finally, we find modules of co-expressed genes that are highly overlapping with previously published modules corresponding to basic cell types and cellular components (Table 4). Such a high level of between-study conformity, particularly regarding differential expression of individual genes, stems from our large sample size and robust statistical methods, adding confidence that our results represent real biological effects.

### Alzheimer's disease involves many cell types

Our results regarding the changing expression patterns of cell type-specific modules suggest that both neurons and glia are affected by AD progression. Specifically, we found that neuron-associated modules showed decreased expression with AD, astrocyte-associated modules showed increased expression with AD, the oligodendrocyte-associated module showed increased expression with age, and a microglia-associated module showed increased expression with Braak stage in controls (Figure 3). While AD is usually thought of as a neurodegenerative disorder, there is mounting evidence that changes in glial cells occur with AD progression as well. Since oligodendrocytes produce the brain's supply of cholesterol and since progression of neurodegeneration in AD follows the reverse pattern of developmental myelination [7], oligodendrocyte dysfunction has been suggested as an early event in AD progression [18], and has been clearly linked to aging [50]. Hundreds of publications have linked astrocytes and microglia to AD progression, generally in the context of inflammation (for example, see [51]), although the complex issue of whether these effects are protective or pathological is still open to debate (reviewed in [52]). Increases in inflammatory markers have been seen in many transcriptional studies of AD [3,4,9,53,54], often occurring early in the disease progression. Finally, both microglia [55] and reactive astrocytes [17] have been found to surround amyloid plaques, suggesting that glial dysfunction, along with neurodegeneration, is something that occurs throughout disease progression.

### Using microglia as a preclinical indicator of Alzheimer's disease pathology

Microglia are extremely sensitive to disease pathologies, and as such could act as diagnostic markers of disease onset or progression [51]. Furthermore, it is widely accepted that microglia often are found near amyloid deposits [15] and that microglia-mediated inflammation contributes to the progression of AD [56]. What association microglia and neuroinflammatory markers have with NFT pathology is less clear. Whereas microglial cell activation has been linked to NFT burden in some cases [57,58], this association has not received nearly the same attention as that of microglia and amyloid plaques [15,59]. Overall, it is clear that microglia activation occurs in the AD brain, but its timing and role in AD progression has been difficult to pin down.

Our finding that microglial markers show increased expression in controls in Braak stage 2 (Figures 3 to 5) lends support to the idea that an increase in inflammatory processes may be one of the earliest events in AD progression. In a longitudinal analysis of blood from approximately 900 subjects, higher protein levels of three inflammatory markers (interleukin 6, α1-antichymotrypsin, and C reactive protein) were associated with an increased risk of dementia in general and of AD specifically [38]. A separate study found that a panel of 18 signaling proteins involved in immune response could accurately predict the transition of mild cognitive impairment to AD when measured in blood plasma [60]. While these are not the same genes that we found differentially expressed with Braak stage, these studies highlight the possibility of using blood biomarkers as a preclinical predictor of AD progression. Immune response genes have also been linked to blood lipid levels [61], another possible indicator of AD progression. Positron emission tomography (PET) is another non-invasive strategy that has the potential of preclinically predicting AD progression. One group found that approximately 40% of the patients they imaged with mild cognitive impairment showed increased microglial activation [62]. Interestingly, the only place where they found significant microglial activation in amyloid-positive versus amyloid-negative mild cognitive impairment patients was frontal cortex, which is consistent with our qRT-PCR validations (Figure 4b). Thus, several studies suggest that some measure of inflammatory markers could be combined with a longitudinal study design to create a relatively accurate predictor of AD onset.

Our results further demonstrate that these same microglial markers show increased expression in or near neurons bearing NFTs (Table S6 in Additional file 6), suggesting that microglia may react to both major AD pathologies, not only amyloid plaques. The major question that remains is whether the upregulation of microglia reflects immune activation, or some other function, such as synaptic pruning or homeostasis [63], and whether this process is protective or dysfunctional. Since microglia can cross the blood brain barrier [64] and since they may be involved in amyloid plaque degradation [59], we surmise that they serve a protective role. At least two studies of transgenic mice with *APP *and *PSEN1 *mutations support this hypothesis. The first found that injection of transgenic mice with macrophage colony-stimulating factor, a protein that stimulates the production of bone marrow-derived microglia, prevents cognitive decline when injected presymptomatically and stabilizes the cognitive decline when injected after the appearance of amyloid pathology [64]. The second study found that activated microglia colocalize with newly formed amyloid plaques within 1 to 2 days, at which point these plaques no longer increase in size, suggesting that microglia may stabilize their growth [15]. Similar results were found in human: in individuals with possible AD, not only were there more microglia and amyloid plaques relative to controls, but amyloid plaques were also never found without an adjacent microglia [16]. Thus, despite the relative lack of success of anti-inflammatory trials to date [65], our results suggest that approaches to AD treatment involving the mobilization of anti-inflammatory processes may have the potential to be both noninvasive and effective.

## Conclusions

Despite a century of study, the number of AD diagnoses continues to increase, suggesting that new strategies for studying AD need to be developed and that previous results need to be confirmed in order to better understand this complex disease. Tollervey and colleagues [66], for example, used splice junction microarrays to find changes in alternative splicing in temporal cortex, both with age and with neurodegenerative disease, allowing them to distinguish disease-specific changes, which mostly affect neurons, from common changes, which affect both neurons and oligodendrocytes. We have taken a complementary approach by confirming previous transcriptional studies of AD on many levels, but go beyond these studies in a number of ways. We find candidate genes for neuroprotection and vulnerability in the AD hippocampus, as well as a robust relationship between disease- and region-specific gene expression changes. We identify co-expression modules corresponding to major cell types, which show expression patterns consistent with known disease-related changes, and suggest that a more detailed look into the role of microglia in preclinical AD is warranted. Together, these results paint a picture of AD as a multifaceted disease involving slight transcriptional changes in many genes between regions, coupled with a systemic immune response, gliosis, and neurodegeneration. Despite this complexity, we find that a consistent picture of gene expression in AD is emerging.

## Abbreviations

AD: Alzheimer's disease; EASE: Enrichment Analysis Systematic Explorer; FC: fold change; GEO: Gene Expression Omnibus; GO: Gene Ontology; NFT: neurofibrillary tangle; PMI: post mortem interval; qRT-PCR: quantitative RT-PCR; TO: topological overlap; WGCNA: weighted gene co-expression network analysis.

## Competing interests

The authors declare that they have no competing interests.

## Authors' contributions

JAM designed the experiments, collected RNA from tissue, analyzed the microarray data, and wrote the paper. RLW collected and prepared tissue for the study and performed follow-up experiments. JMG performed experiments confirming results from the microarray analysis. SH designed several microarray analysis methods used in this study and provided general statistical guidance. DHG helped design the experiment, provided guidance on all aspects of the project, and helped write the paper. All authors read and approved the final manuscript.

## Supplementary Material

Additional file 1 **Table S1**. Phenotypic information for each subject used in this study. Click here for file

Additional file 2 **Table S2**. All differentially expressed genes across all comparisons. Click here for file

Additional file 3 **Table S3**. Statistics comparing CA1 versus CA3 expression in the Allen Human Brain Atlas [40]. Click here for file

Additional file 4 **Table S4**. All genes that are both disease-altered and region-enriched. Click here for file

Additional file 5 **Table S5**. Module membership values for each gene and its assigned module from the WGCNA. Click here for file

Additional file 6 **Supplementary Figures S1 to S7 and Tables S6 and S7**. Figure S1 shows that there are no obvious confounding factors in our data. Figure S2 plots the number of differentially expressed genes for each comparison. Figure S3 plots common region-enriched genes between this study and [40]. Figure S4 shows the agreement between disease-altered genes in this study and [3]. Figure S5 shows *in situ *hybridization validation for UNC13C in human brain. Figure S6 shows that around half of differentially expressed genes are due to changes in cell type composition. Figure S7 shows the network depictions and module assignments for the WGCNA. Figure S8 plots the top genes and connections for each module in the WGCNA. Table S6 lists the top 25 NFT-associated genes (of which 20 are in a microglial-associated module). Table S7 lists the primer pairs used for qRT-PCR validation. Click here for file
