# Supplementary material for: Genes and pathways underlying regional and cell type changes in Alzheimer's disease
Source: Genome Med. 2013 May 25;5(5):48. doi: 10.1186/gm452 (PMC3706780; doi:10.1186/gm452)
Supplement: Additional file 6 — Supplementary Figures S1 to S7 and Tables S6 and S7. Figure S1 shows that there are no obvious confounding factors in our data. Figure S2 plots the number of differentially expressed genes for each comparison. Figure S3 plots common region-enriched genes between this study and [40]. Figure S4 shows the agreement between disease-altered genes in this study and [3]. Figure S5 shows in situ hybridization validation for UNC13C in human brain. Figure S6 shows that around half of differentially expressed genes are due to changes in cell type composition. Figure S7 shows the network depictions and module assignments for the WGCNA. Figure S8 plots the top genes and connections for each module in the WGCNA. Table S6 lists the top 25 NFT-associated genes (of which 20 are in a microglial-associated module). Table S7 lists the primer pairs used for qRT-PCR validation. [file gm452-S6.PDF]

Supplementary Information: Table of Contents

Table of Contents ------------------------------ 1
Supplementary Figures ------------------------- 2
Supplementary Figure 1 -- 2
Supplementary Figure 2 -- 3
Supplementary Figure 3 -- 4
Supplementary Figure 4 -- 5
Supplementary Figure 5 -- 6
Supplementary Figure 6 -- 7
Supplementary Figure 7 -- 8
Supplementary Figure 8 -- 9
Supplementary Tables -------------------------- 28
Supplementary Tables 1-5 ------------------- (in separate files)
Supplementary Table 6 -- 28
Supplementary Table 7 -- 29

Supplementary Figure 1

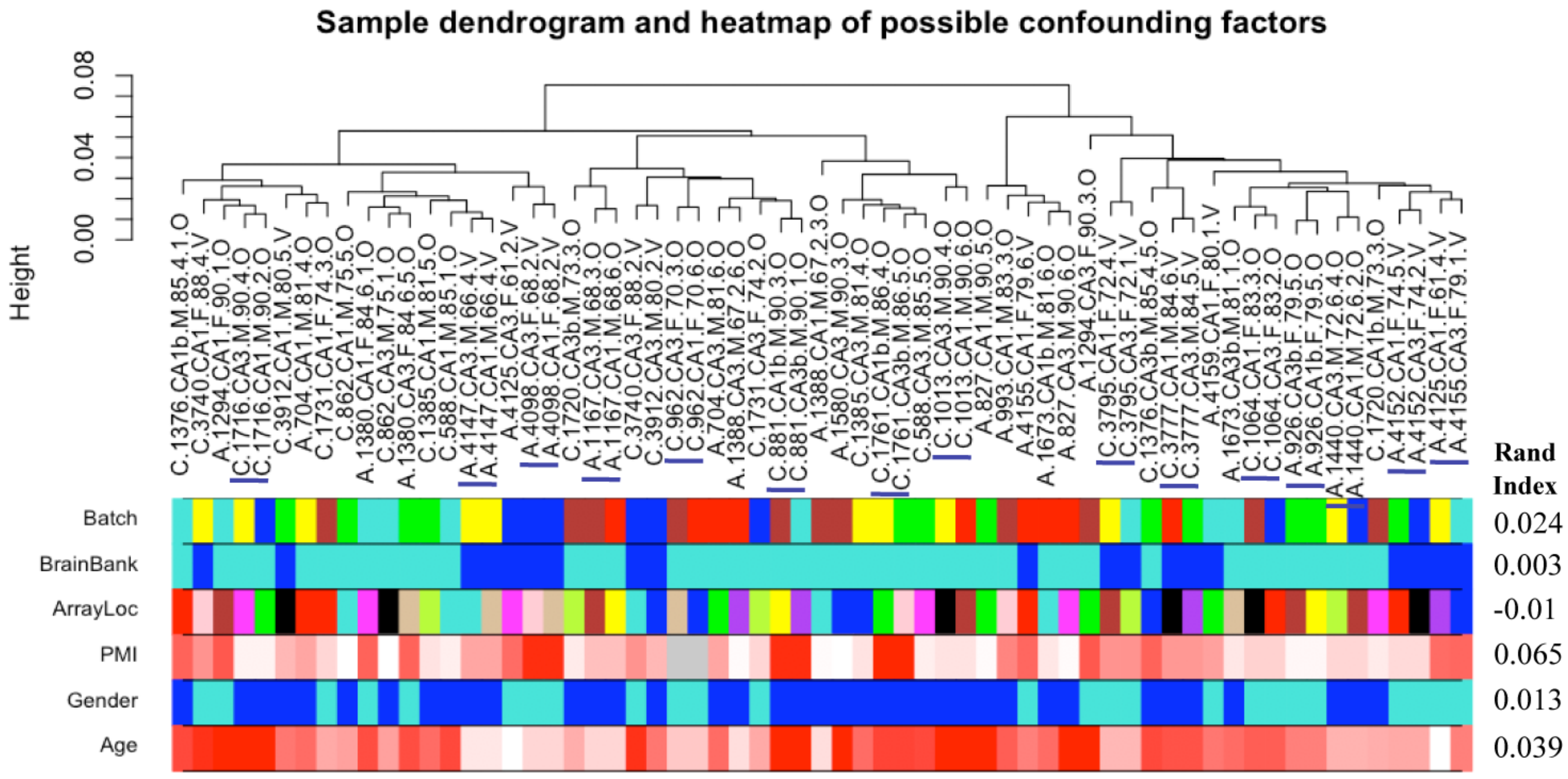

Supplementary Figure 1: There are no obvious confounding factors in our data. Top trace: Dendrogram plotting all samples using hierarchical clustering across all genes. Paired regional samples from the same subject often cluster together (blue horizontal bars), which is to be expected. Bottom traces: sample information is displayed such that each color represents a different value for the following phenotypes: Batch (Illumina chip), BrainBank, ArrayLoc (position on chip), PMI, Gender, and Age. Rand indices near zero indicate that a there are no obvious groupings of samples along any of these phenotypes.

Supplementary Figure 2

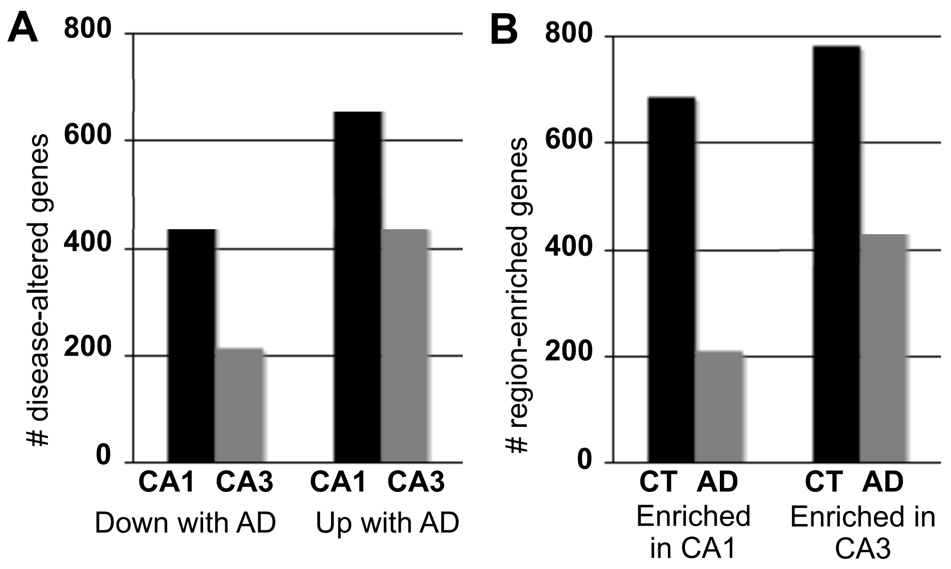

Supplementary Figure 2: Number of differentially expressed genes for each comparison. A) Fewer genes are disease-altered in CA3 (grey bars) than in CA1 (black bars), consistent with this region's relative neuroprotection. B) Fewer genes are region-enriched in AD (grey bars) than in controls (black bars), consistent with studies of ischemia. Y-axes show the number of differentially expressed genes and x-axes show trait information.

Supplementary Figure 3

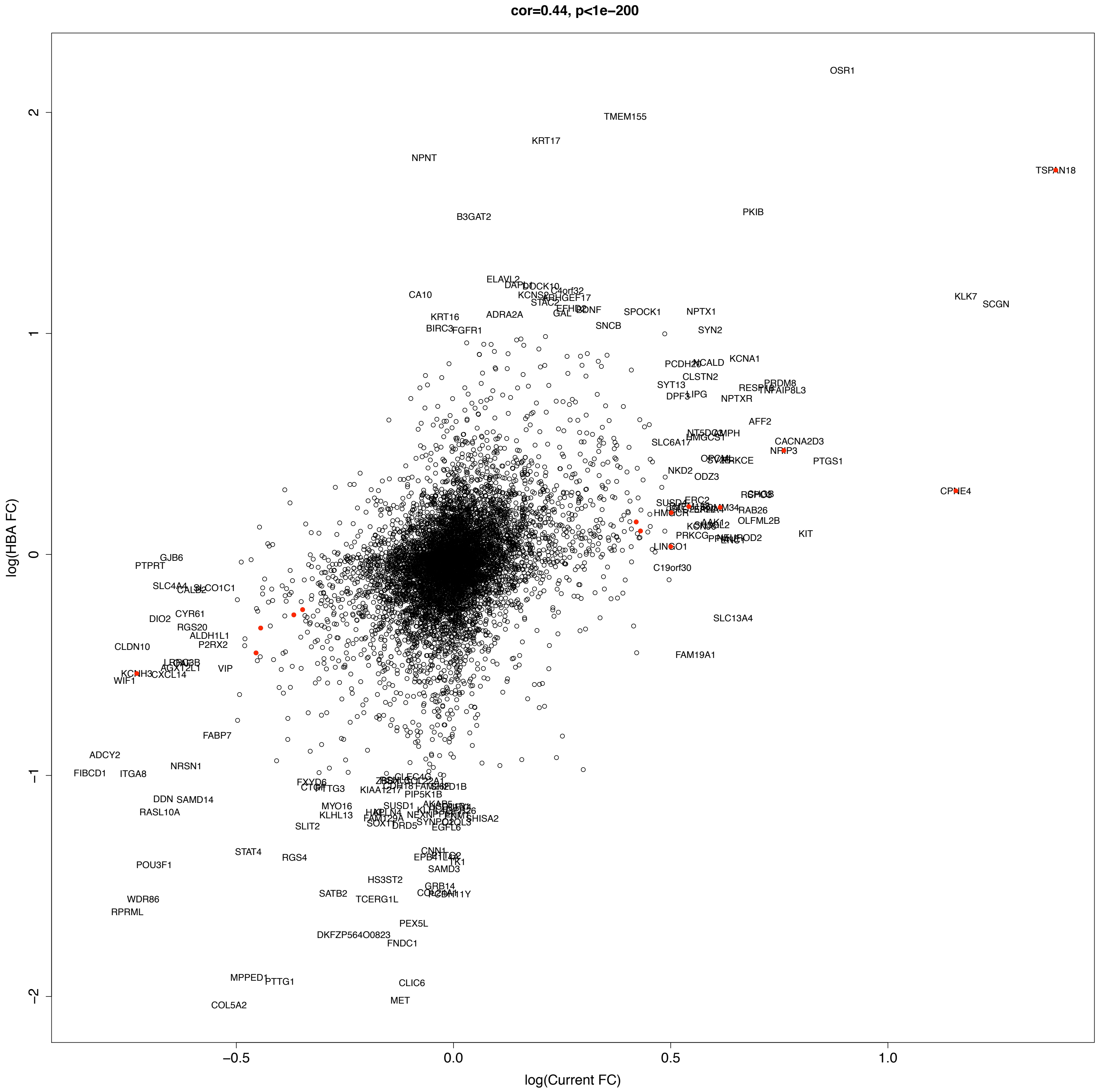

Supplementary Figure 3: Common region-enriched genes in this study and in a new human brain atlas. These graphs plot the correlation of region-specificity (as measured by the log of the ratio of the average CA3 vs. CA1 expression) in the 14010 common genes between this study (x-axis) and the Allen Human Brain Atlas (y-axis) (http://human.brain-map.org/). Each dot represents a gene. Results are highly consistent between data sets as measured both by Pearson correlation (R) and p-value. Genes in the upper right (CA3-specific in both studies) and lower left (CA1-specific in both studies) are highlighted as genes that can more confidently be called region-specific than genes that are region-enriched in a single study.

Supplementary Figure 4

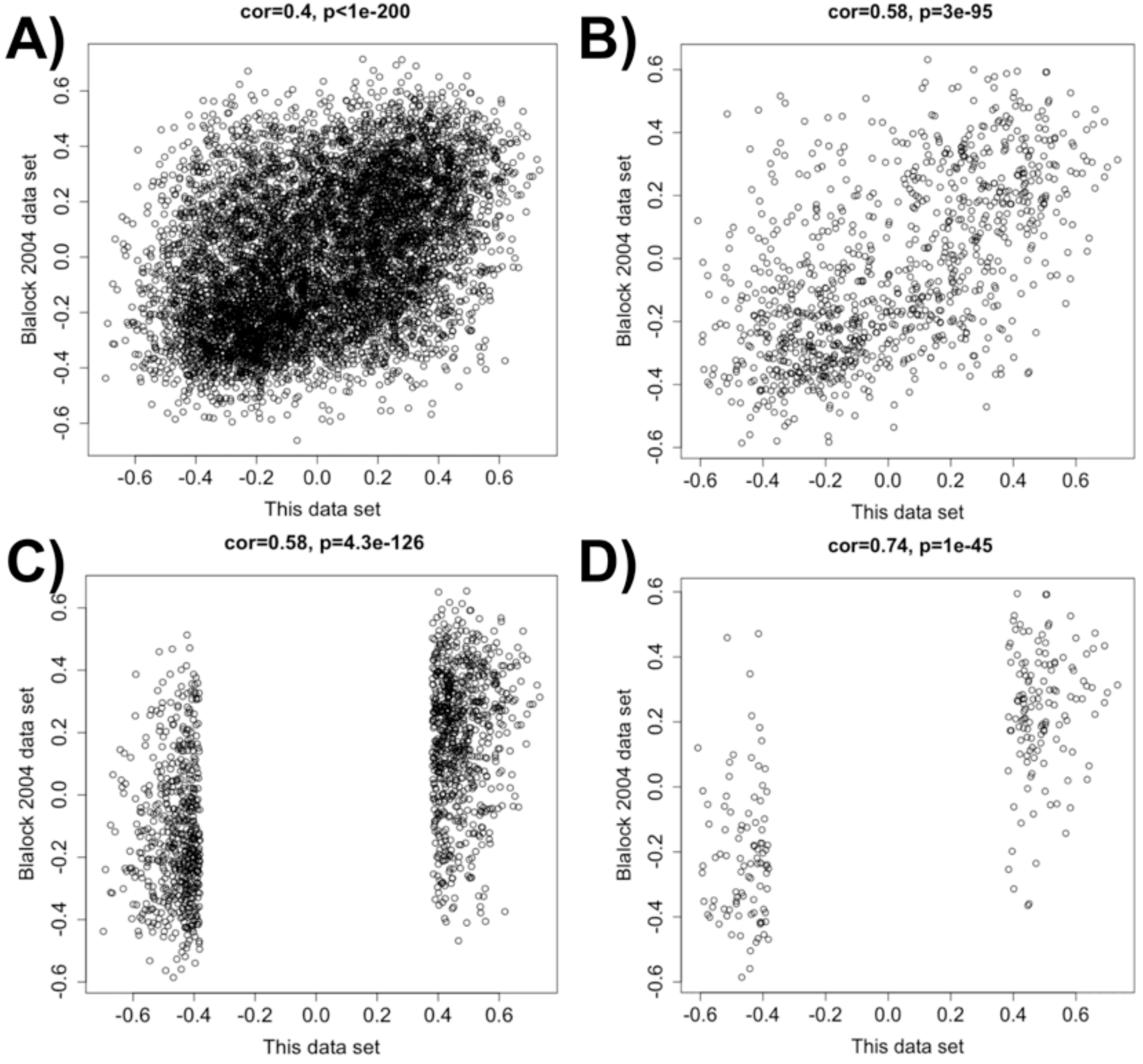

E)

Gene	Worst Corr.	Gene	Worst Corr.
TGFBR3	0.57	SH3GLB2	-0.53
NXT1	0.56	IQSEC2	-0.49
GNA12	0.54	PTN	-0.47
AHNAK	0.53	EPB49	-0.46
PRKX	0.53	GABRB3	-0.46
C1S	0.52	COX4I1	-0.46
GMPR	0.51	GAD1	-0.45
RHOQ	0.51	NRXN1	-0.44
ITSN1	0.51	COX6A1	-0.44
SPEN	0.50	TUBG2	-0.44
ZFP106	0.50	CNNM1	-0.44
MYBPC1	0.50	NIT1	-0.44
NFE2L1	0.50	**KCNIP1**	-0.44
SMAD2	0.49	PH-4	-0.44
EPOR	0.49	WIF1	-0.44
STAG2	0.49	ETFB	-0.43
POLR2H	0.49	CENTG1	-0.43
MORC4	0.49	GOLGA8B	-0.43
FNBP1L	0.48	ATG7	-0.42
PHF17	0.48	C13orf1	-0.42
MCL1	0.48	FH	-0.42
FLII	0.47	OMG	-0.42
PDE4DIP	0.47	NEDD8	-0.42
**DMN**	0.47	PENK	-0.42
IL20RA	0.47	MAPRE3	-0.42

Supplementary Figure 4: In disease, differential expression for highly expressed and highly differentially expressed genes is more reliable between studies. These graphs plot the correlation between disease association in the 6950 common genes between this study and a previous study of AD progression (Blalock et al 2004). The x-axes represent correlation between gene expression and Braak score in this data set, while the y-axes represent the same measure in the Blalock data set. Each dot represents a gene. Results are highly consistent between data sets as measured both by Pearson correlation (R) and p-value (A). Note that by including only genes with high expression (B) or significant genotype/phenotype correlation (C), we improve our correlations. The results are even better if we use both filters (D). Note that the p-values become less significant as the correlations get higher due to a decrease in the number of genes in each analysis. E) Top 25 genes positively and negatively correlated with NFT burden in both studies. Genes were sorted by the least significant correlation (Worst corr.) between studies. Genes from table 3 are presented in bold.

Supplementary Figure 5

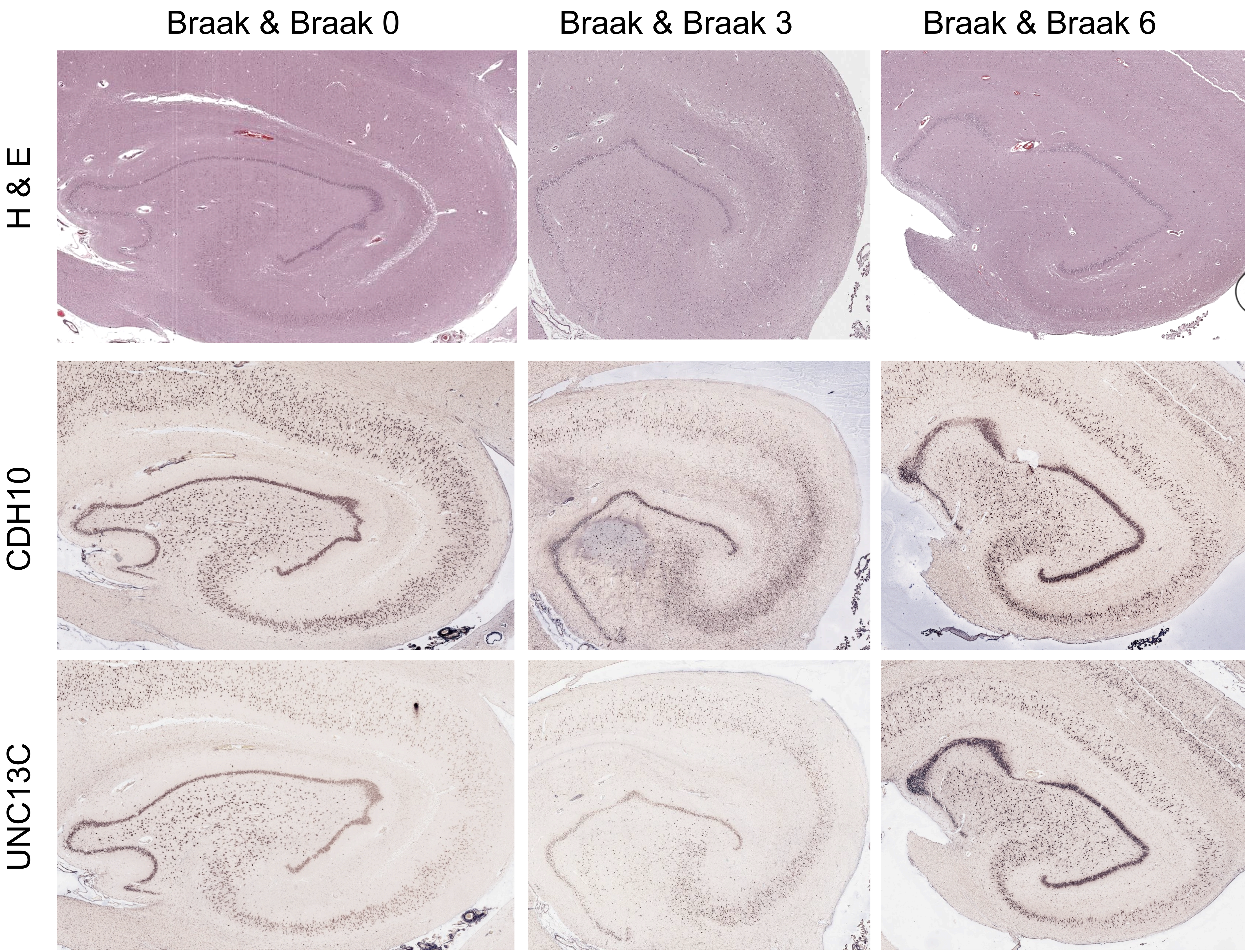

Supplementary Figure 5: Confirmation of UNC13C in control (left), moderate (center), and severe (right) AD cases individuals using RNA in situ hybridization. Braak & Braak staging of individuals is indicated above each column. Top: hematoxylin/eosin staining. Center: CDH10, positive control gene expressed evenly across CA3 and CA1. Bottom: UNC13C, a putative protection gene, with increased expression in CA3 relative to CA1, which is stable in control and AD patient brain.

Supplementary Figure 6

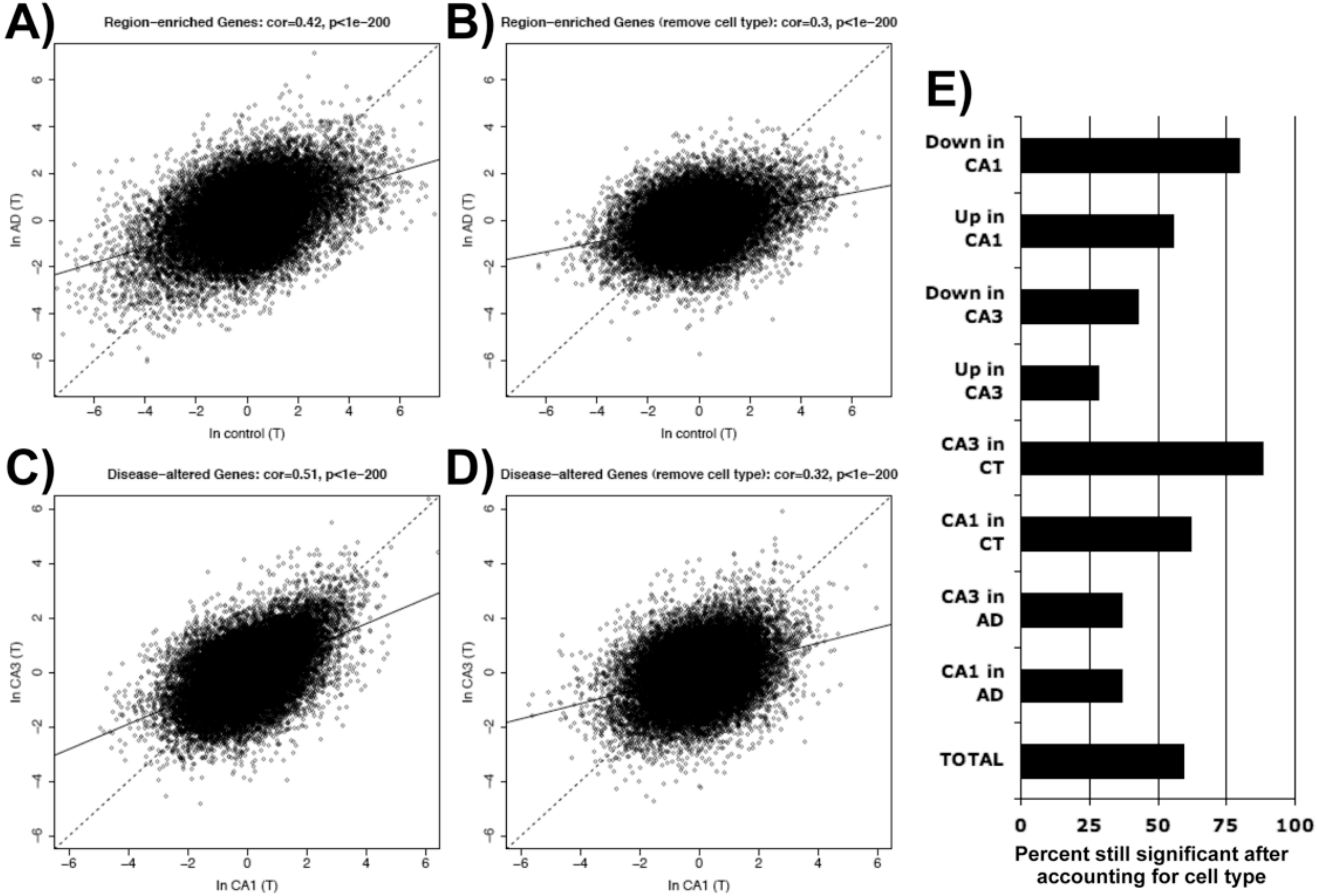

Supplementary Figure 6: Around half of the differentially expressed genes are due to changes in cell type composition. A-B) Plot of correlations between T-scores of region-enriched genes in control vs. AD samples. Both before (A) and after (B) accounting for cell type using a linear model, there is significant correlation, but the correlation is decreased after accounting for cell type. C-D) Plot of correlations between T-scores of disease-altered genes in CA1 vs. CA3. Both before (C) and after (D) accounting for cell type using a linear model, there is significant correlation, but the correlation is decreased after accounting for cell type. E) Bar graphs showing the percent of differentially expressed genes still significant after accounting for cell type in each of the 8 categories. In total, around half of the differentially expressed genes are due to changes in cell type composition.

Supplementary Figure 7

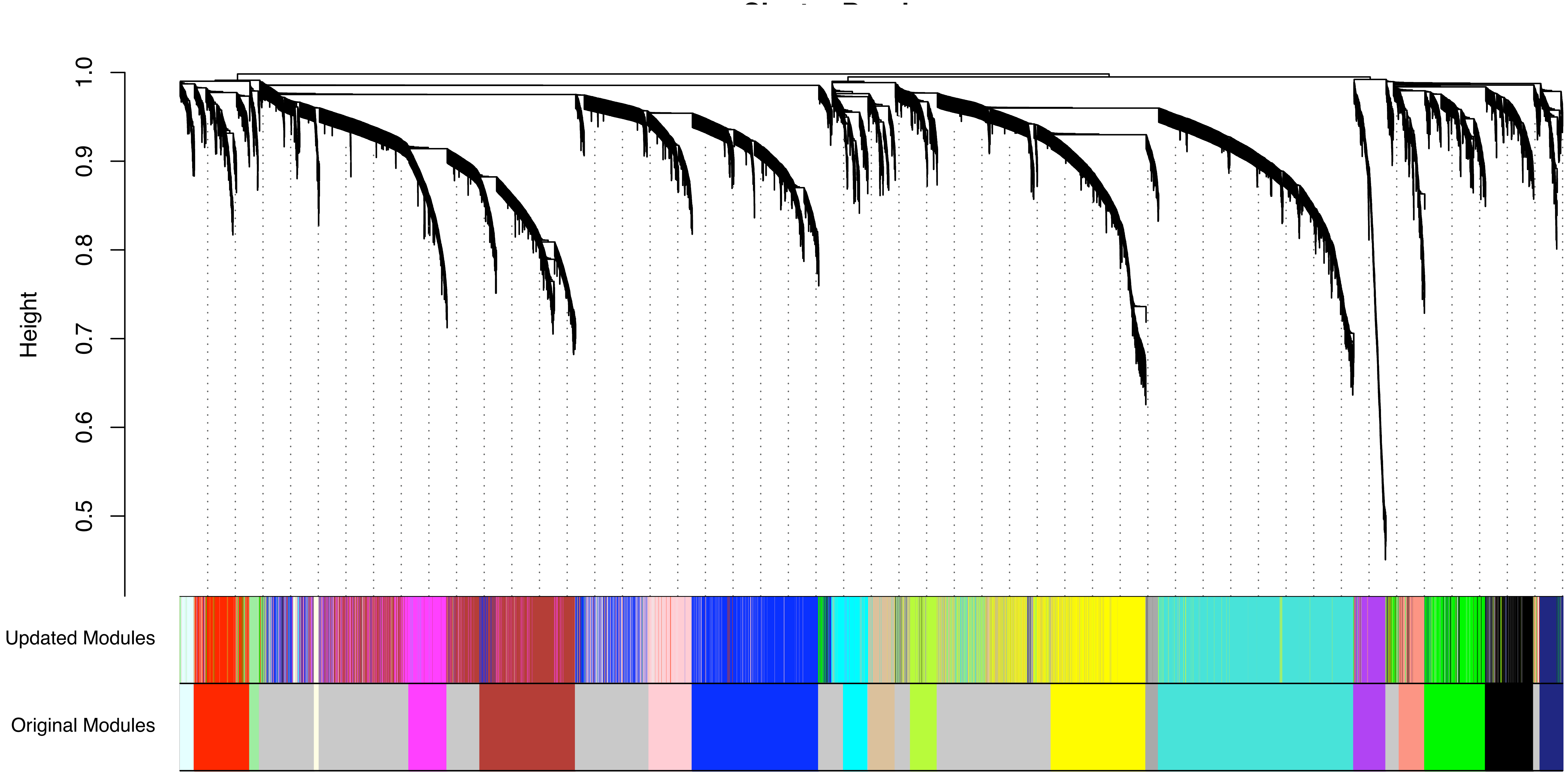

Supplementary Figure 7: Network depiction and module assignments. Cluster dendrogram of genes in the gene expression network (top trace). The y-axis corresponds to topological distance (1-TO). Original module assignments for 19 modules were made using a dynamic tree cutting algorithm (bottom trace). Final (updated) module assignments were made by reassigning each gene to the module with which it has the highest module membership (middle trace).

Supplementary Figure 8

(This figure spans the next 18 pages)

Supplementary Figure 8: Network depictions of modules allow visualization of within-module connections and hub genes. For each module (A=black, B=blue, C=brown, D=cyan, E=green, F=greenyellow, G=grey60, H=lightcyan, I=lightyellow, J=magenta, K=midnightblue, L=pink, M=purple, N=red, O=salmon, P=tan, Q=turquoise, and R=yellow) the top 250 gene-gene interactions are displayed as measured by TO. Large, labeled nodes (genes) represent hub genes with at least 15 connections. The length of each line and the position of each node were arbitrarily chosen by VisANT to highlight network structure. To the right of each network depiction, the top 25 genes for that module are presented (in descending order by module membership). Network depictions of the lightgreen module are presented in Figure 5.

Figure S8A (black)

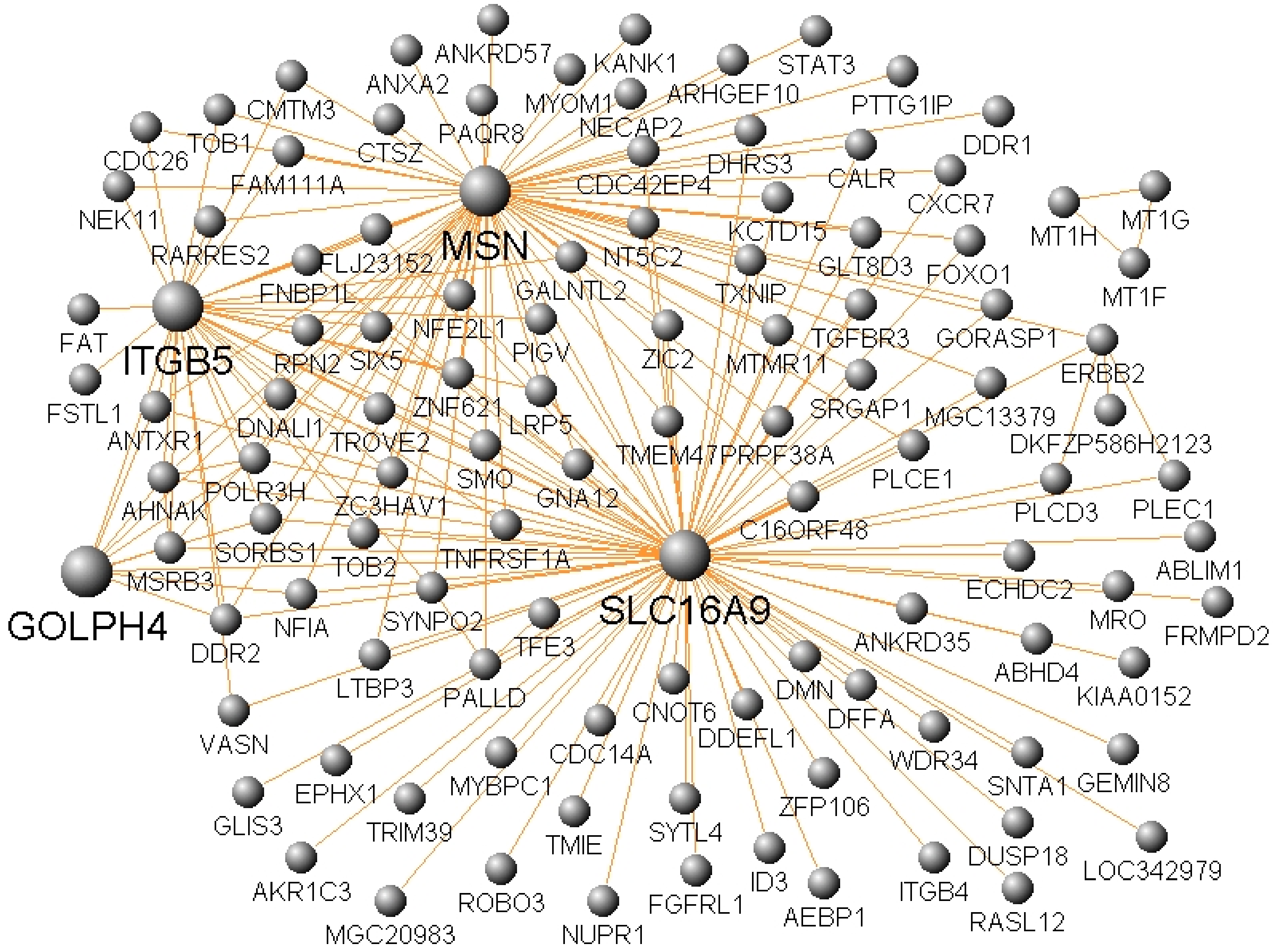

Top Genes
MSN
SLC16A9
ITGB5
TOB2
ARHGEF10
STK3
MGC13379
GOLPH4
TROVE2
PTTG1IP
TNFRSF1A
ZNF621
LTBP3
AHNAK
LRP10
ZC3HAV1
CDC42EP4
PLCE1
NFIA
ERBB2
CDC26
MTMR11
TXNIP
DHRS3
PALLD

Figure S8B (blue)

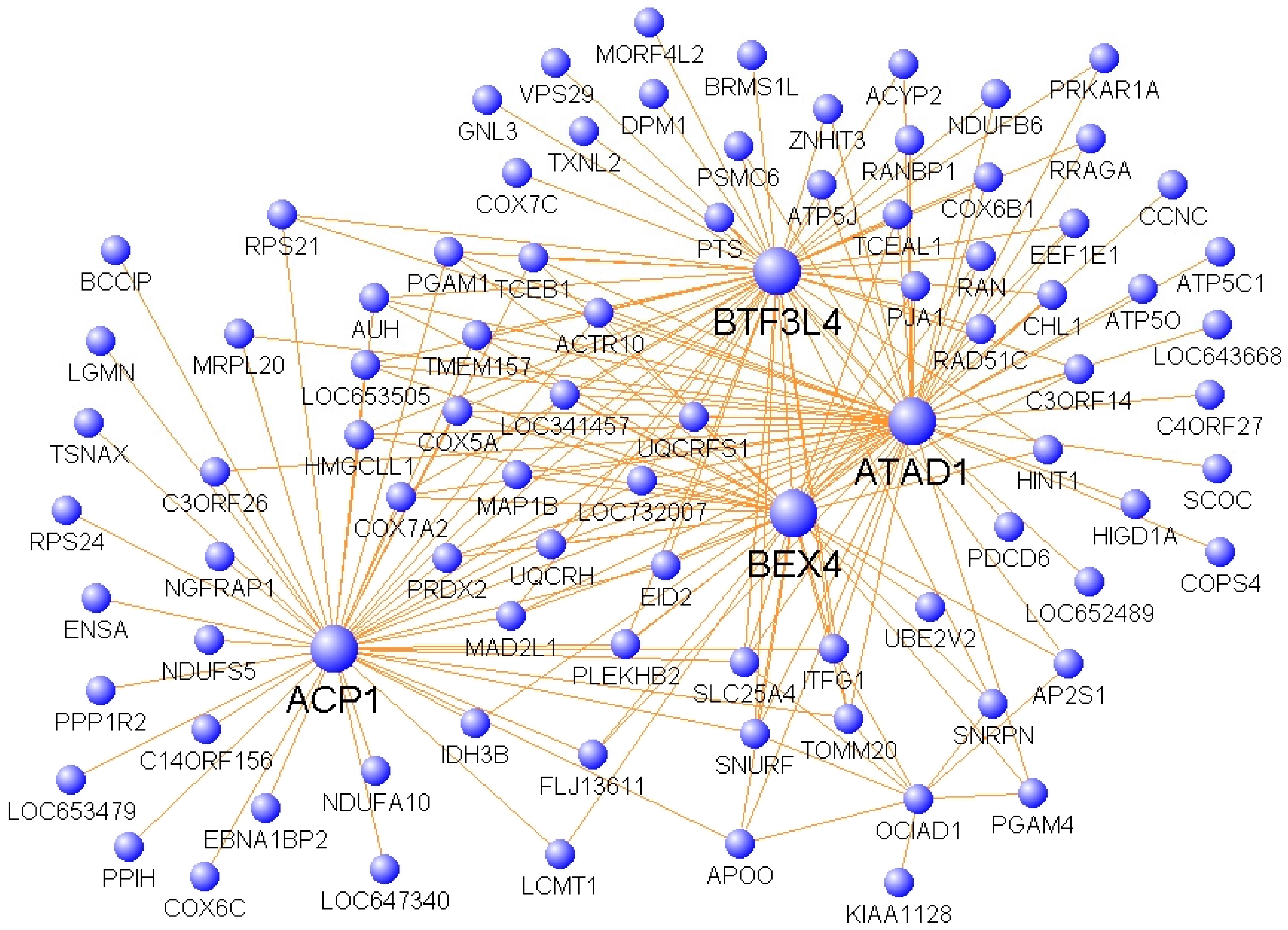

Top Genes
ATAD1
KIAA1279
BTF3L4
BEX4
POLR2B
NAT5
ATP5C1
ACP1
SCOC
LOC653147
PSMD6
HIGD1A
TMEM126B
RRAGA
CHMP2B
ACTR10
PSMA1
OCIAD1
THYN1
COPS4
DNAJC19
ATP5F1
PSMA5
EEF1E1
PRNP

Figure S8C (brown)

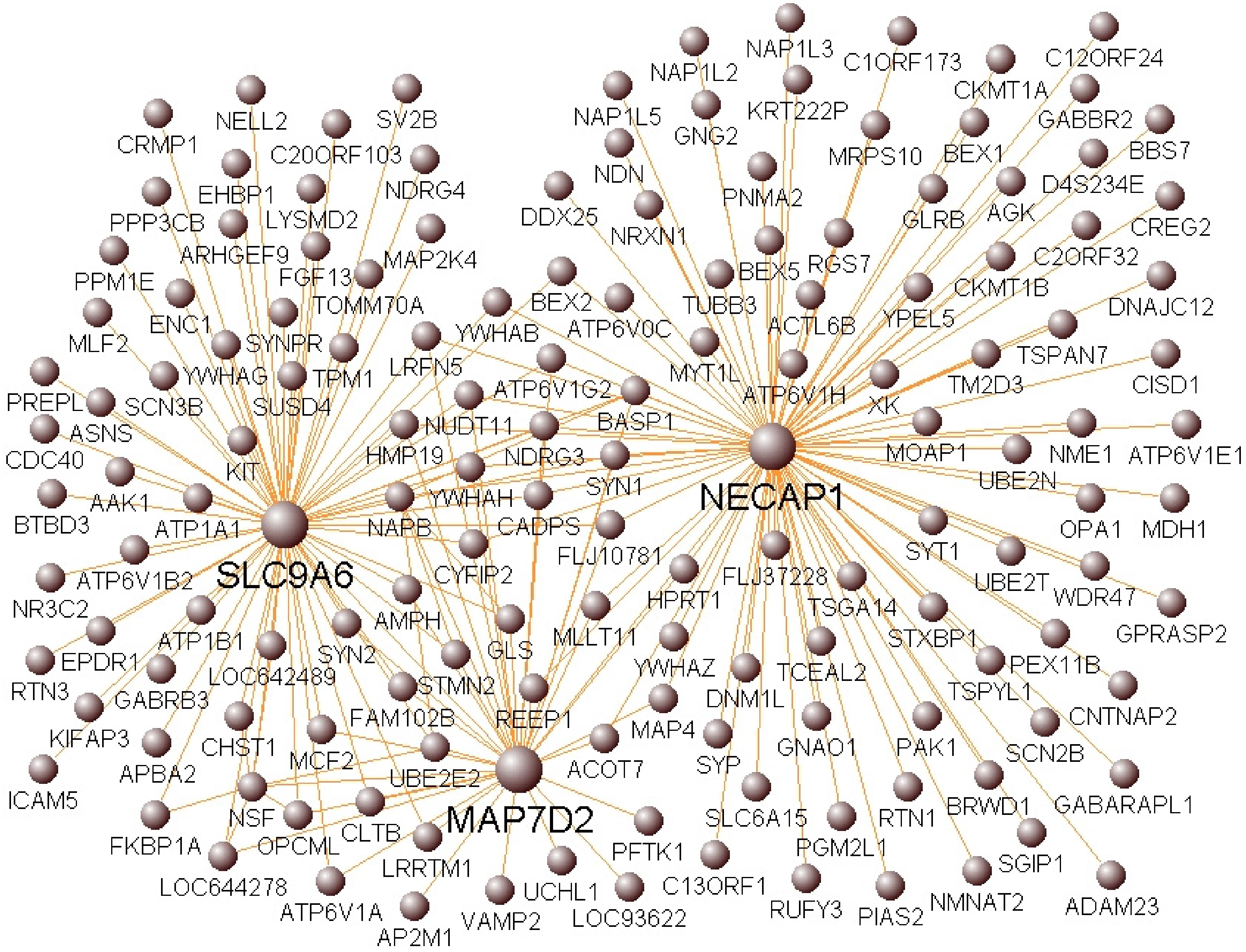

Top Genes

SLC9A6
MAP7D2
WDR7
NECAP1
PGM2L1
PFTK1
NSF
CADPS
NAPB
NMNAT2
NELL2
SULT4A1
LRFN5
HPRT1
CYFIP2
BEX2
CHN1
MAP2K4
GPRASP2
LRRTM1
YWHAB
BEX1
PREPL
MAP4
FGF13

Figure S8D (cyan)

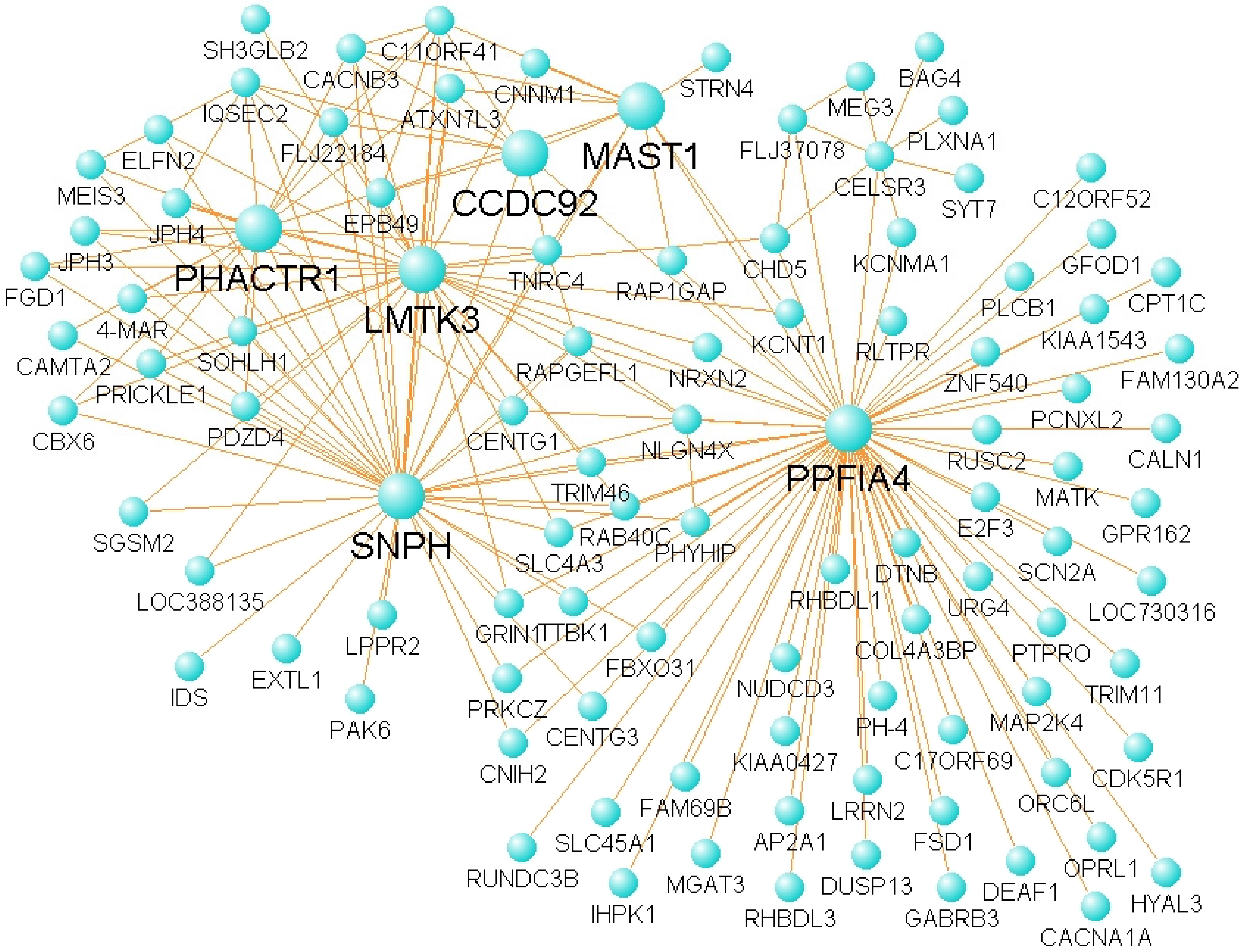

Top Genes
PPFIA4
FLJ37078
SNPH
CELSR3
GFOD1
PRKCZ
FSD1
RUSC2
LMTK3
CALN1
MGAT3
CHD5
FAM130A2
CENTG3
KCNMA1
MAST1
PHYHIP
RAPGEFL1
FAM69B
GRIN1
PCNXL2
BAG4
SCN2A
NUDCD3
MATK

Figure S8E (green)

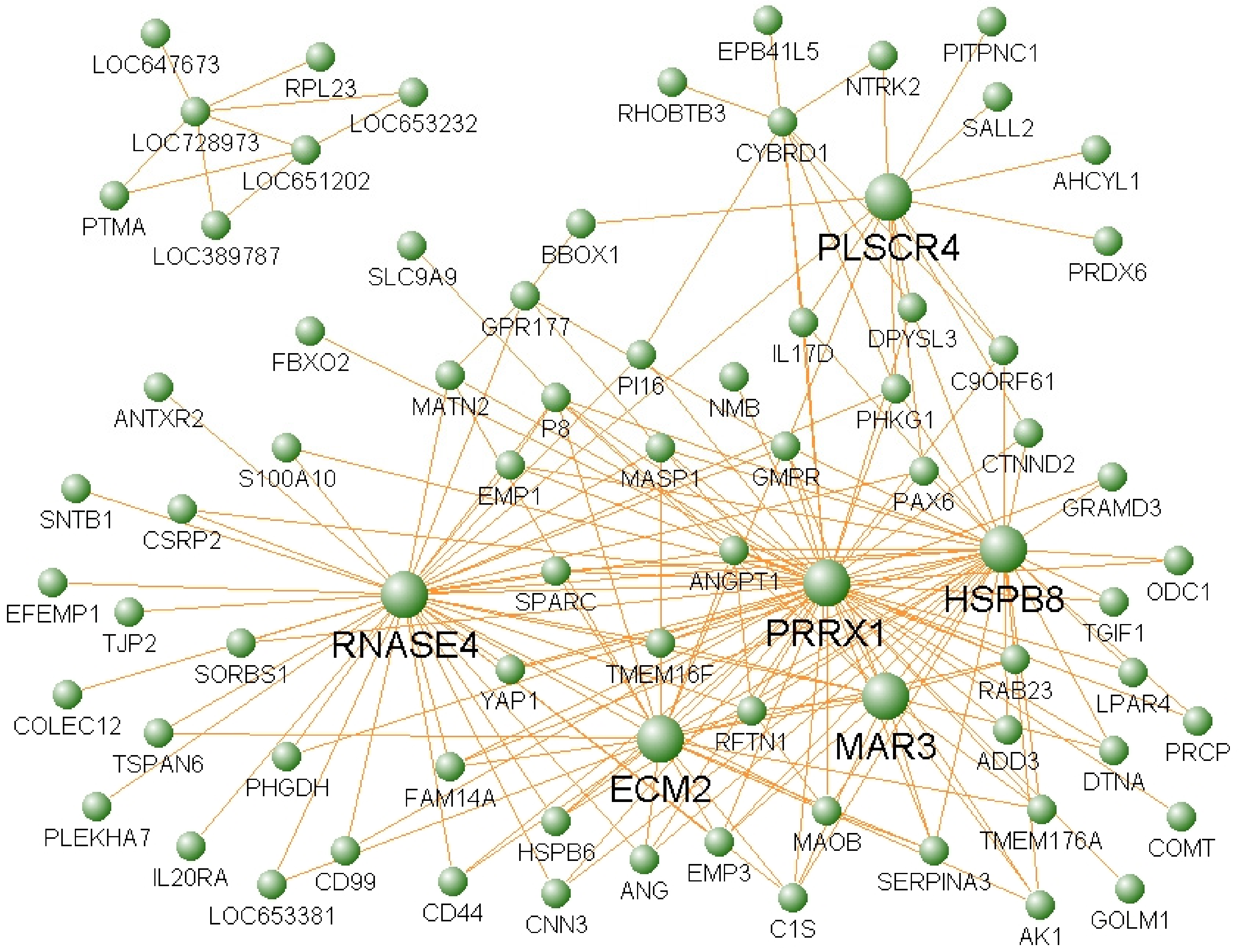

Top Genes

RNASE4
SYNC1
SLC9A9
PLSCR4
MGST1
PRRX1
GPR177
MAR3
GNA13
CYBRD1
GJA1
SNTB1
SLC14A1
NEBL
EMP1
HSPB8
YAP1
CCDC109B
CAT
ANTXR2
ECM2
CSRP2
EFEMP1
PDLIM3
TMED5

Figure S8F (greenyellow)

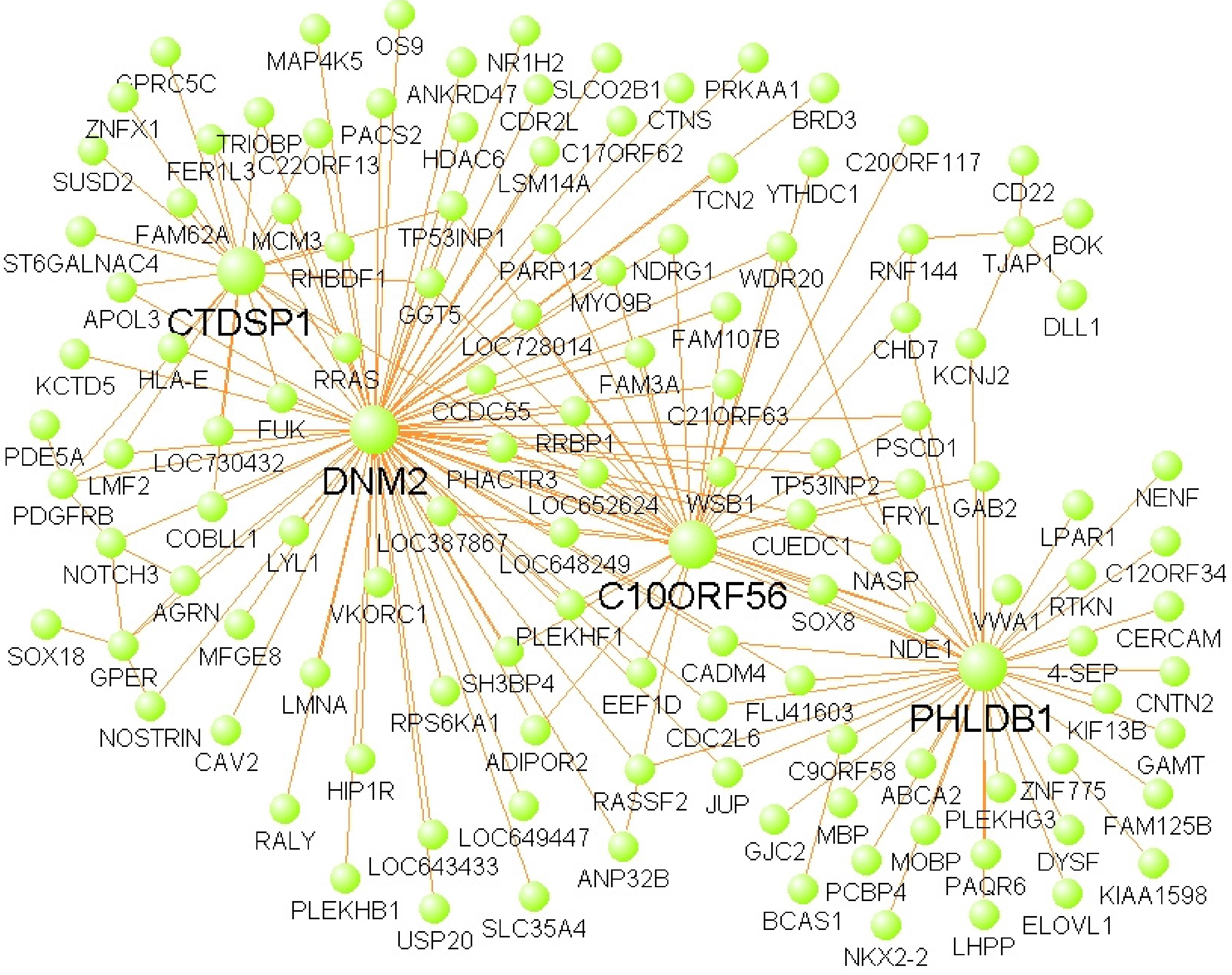

Top Genes

PHLDB1
DNM2
C10orf56
CTNS
RRBP1
KIF13B
PCBP4
CDC2L6
CTDSP1
C17orf62
TRIOBP
RALY
LOC374395
CHD7
RRAS
VWA1
PLSCR3
CUEDC1
CADM4
LOC644511
PSCD1
FAM3A
GAB2
FAM125A
YTHDC1

Figure S8G (grey60)

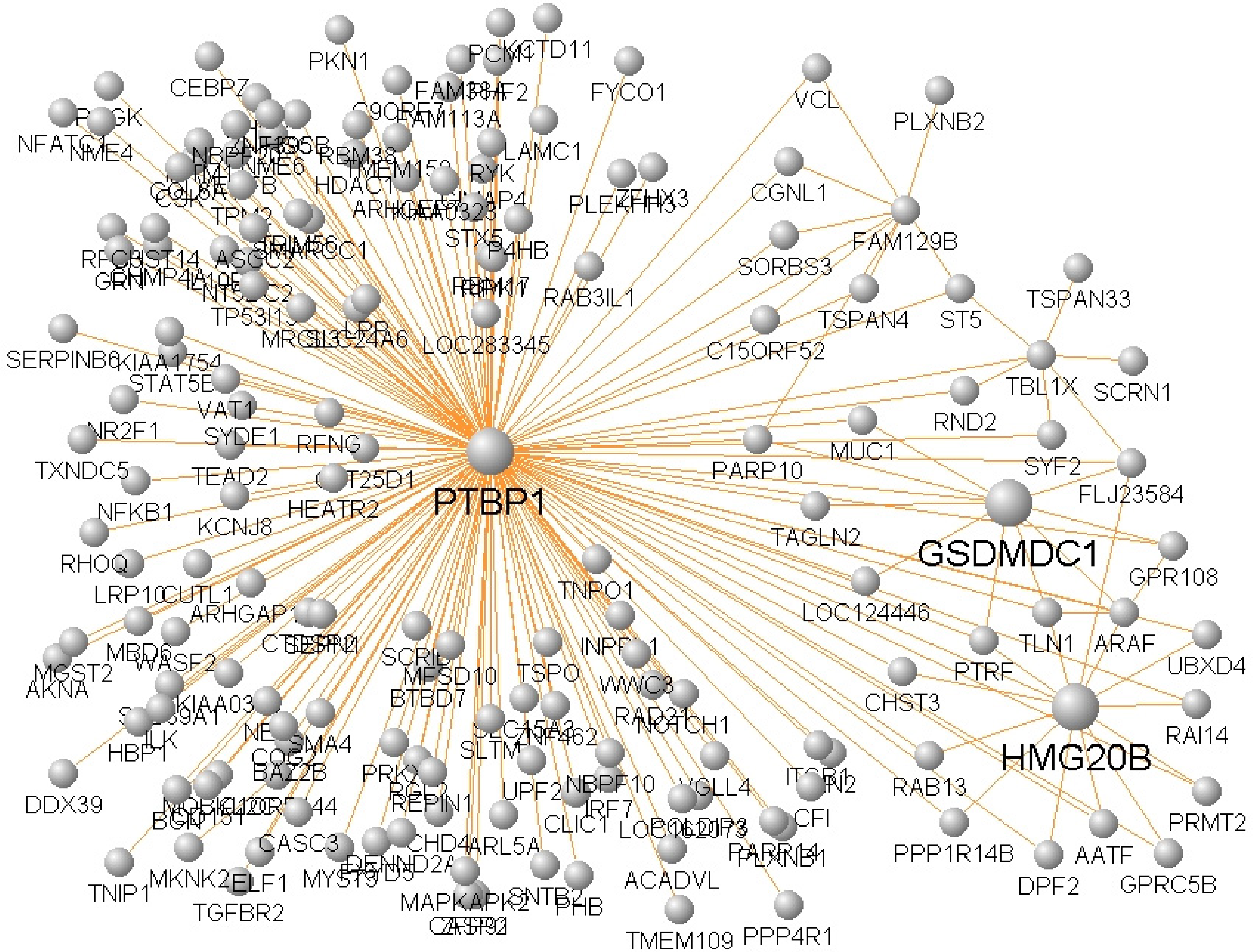

Top Genes
PTBP1
FAM129B
TBL1X
HMG20B
GSDMDC1
UBXD4
ARAF
FYCO1
LPP
MOBKL2C
TAGLN2
LRP10
FAM38A
RHOQ
LAMC1
MYST3
PPP4R1
TMEM150
MBD6
TSPAN33
WASF2
CD151
ZFHX3
CASC3
CSK

Figure S8H (lightcyan)

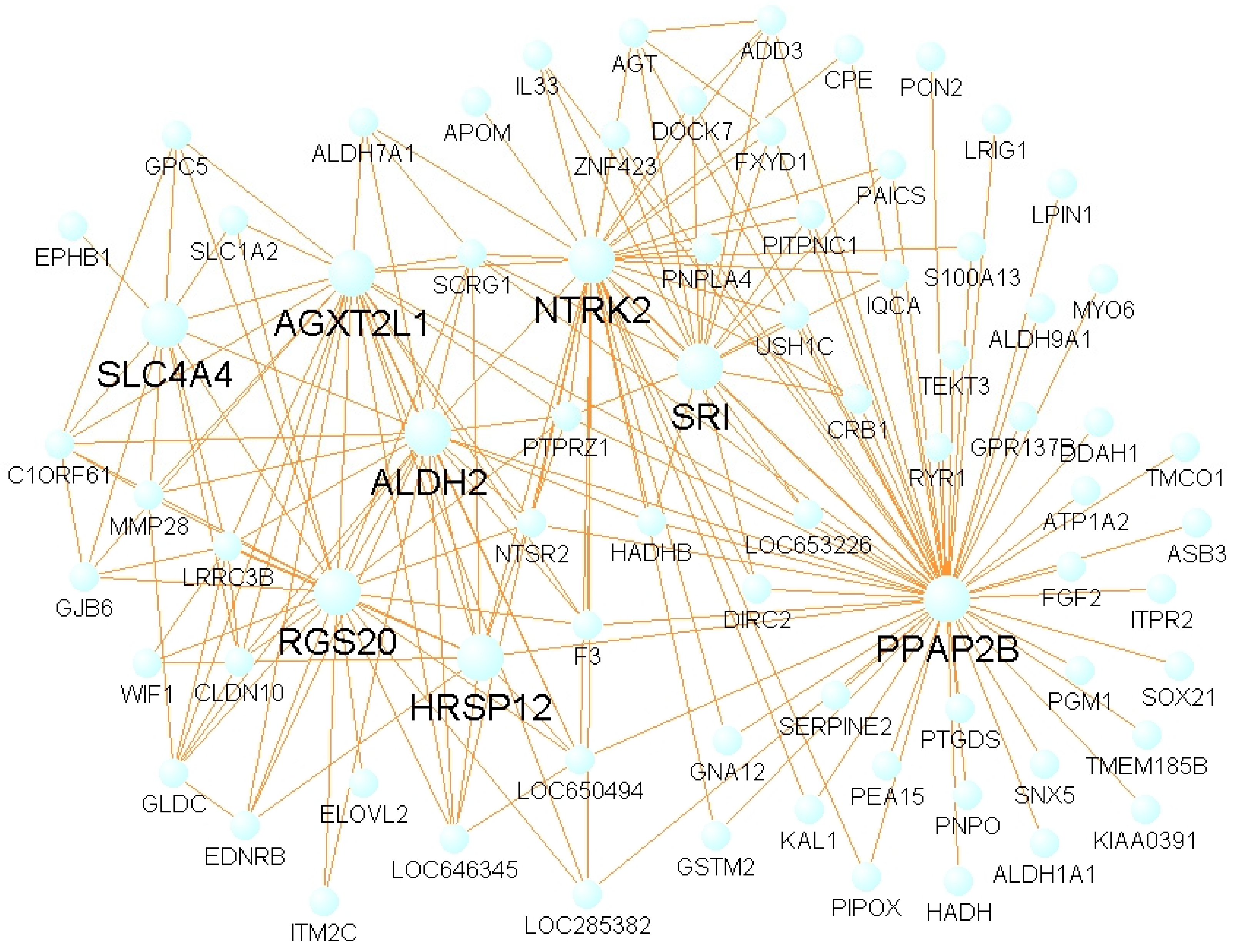

Top Genes

PPAP2B
ALDH2
DOCK7
NTRK2
SRI
SERPINE2
ALDH9A1
PECI
AGXT2L1
HRSP12
RGS20
IL33
AGT
ZNF423
PAICS
LOC653226
ALDH7A1
GLDC
GSTM2
SRP9
DDAH1
SPARCL1
ITM2C
HADHB
F3

Figure S8I (lightyellow)

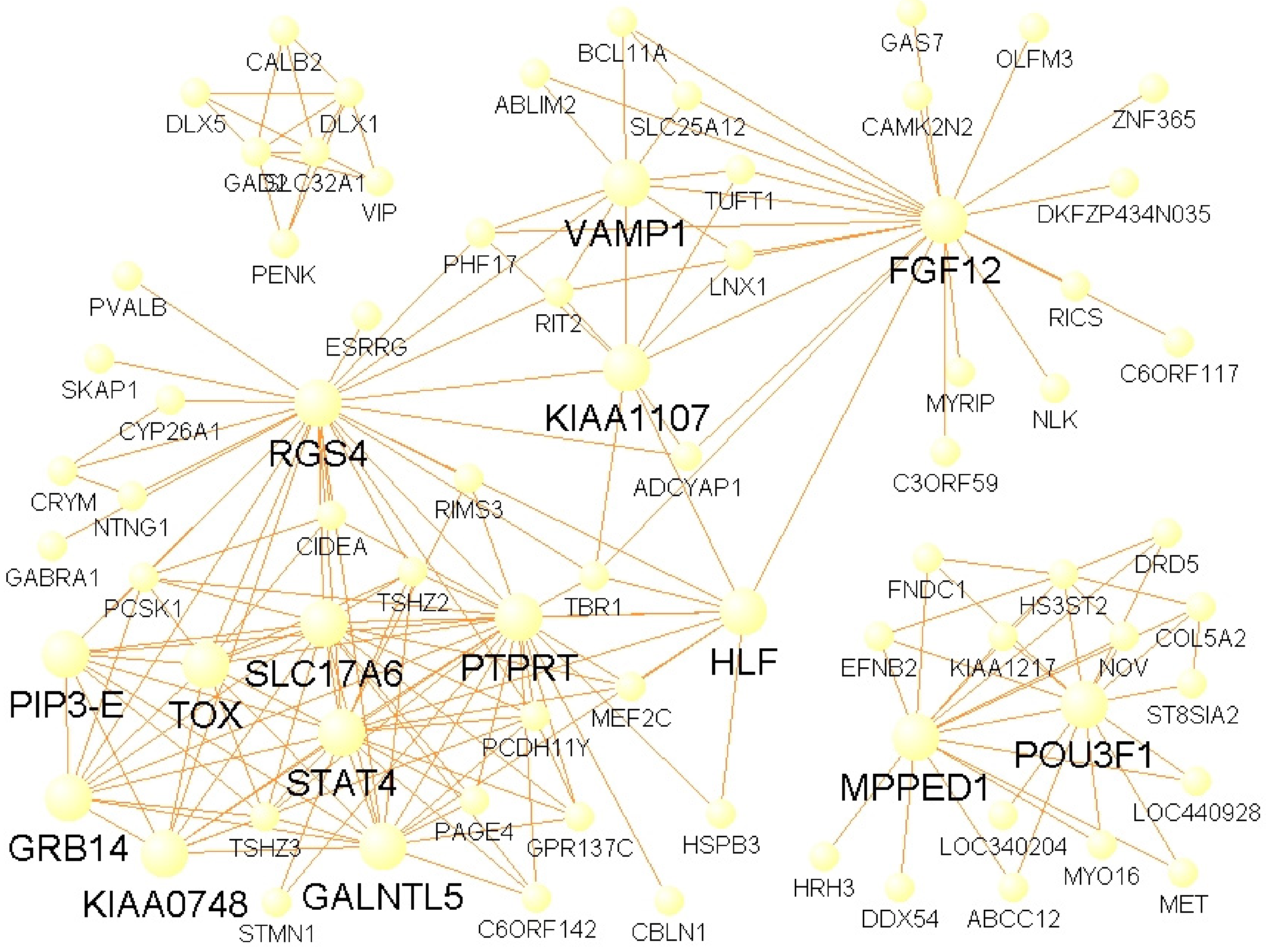

Top Genes
RGS4
KIAA1107
PCSK1
NRSN1
GPR137C
PTPRT
CRYM
HLF
PVALB
TOX
GRB14
STAT4
VAMP1
FGF12
GABRA1
CIDEA
PCDH11Y
DPP10
SLC25A12
SLC17A6
NGEF
OLFM3
PIP3-E
ESRRG
SKAP1

Figure S8J (magenta)

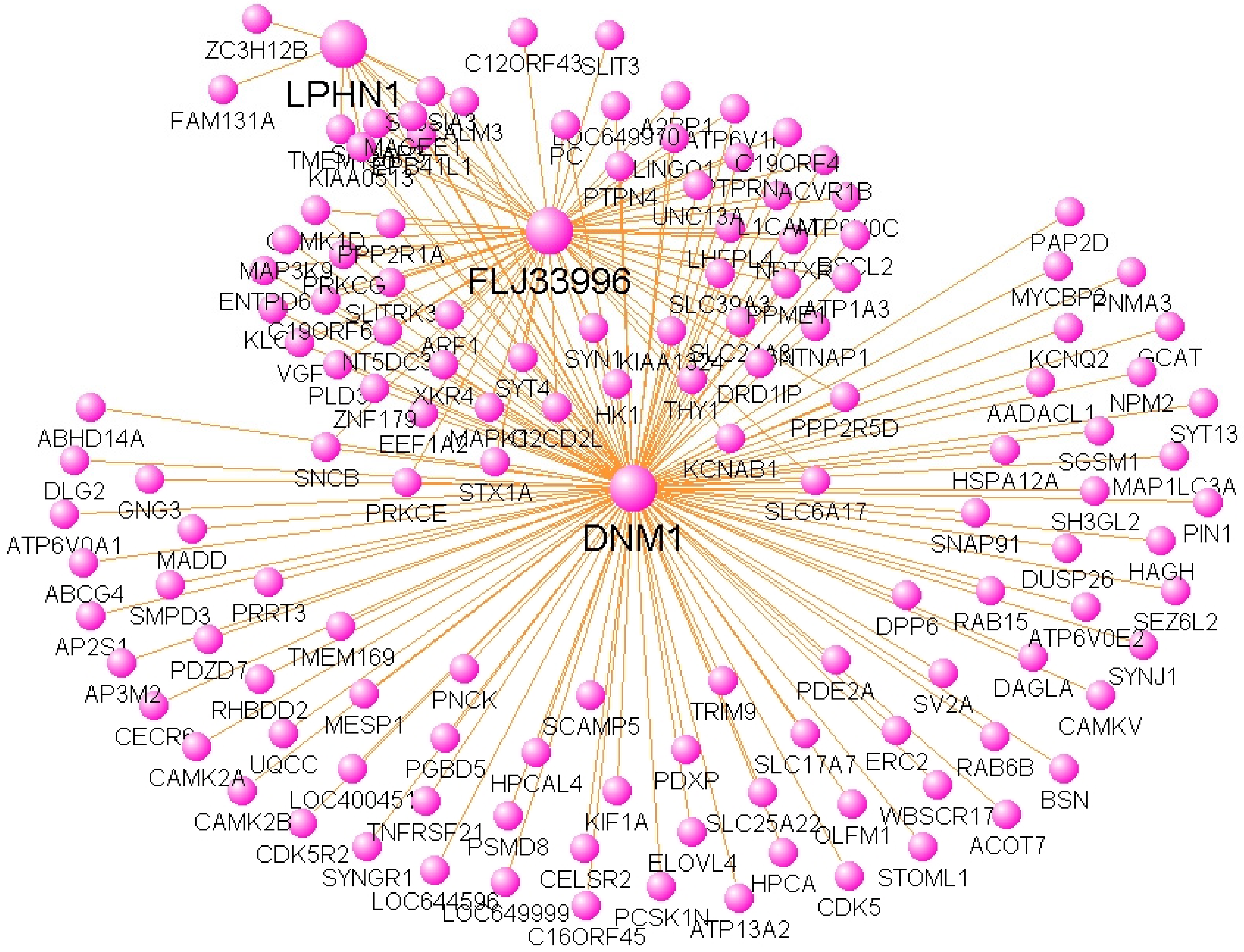

Top Genes

DNM1
THY1
BSN
ELOVL4
FLJ33996
LPHN1
ABCG4
WBSCR17
KCNJ6
LOC649970
SYN1
CACNA1E
C19orf4
EEF1A2
DAGLA
RYR2
SCAMP5
PNMA3
SNAP91
PAP2D
TOMM34
SLC6A17
LHFPL4
CNTNAP1
SLITRK3

Figure S8K (midnightblue)

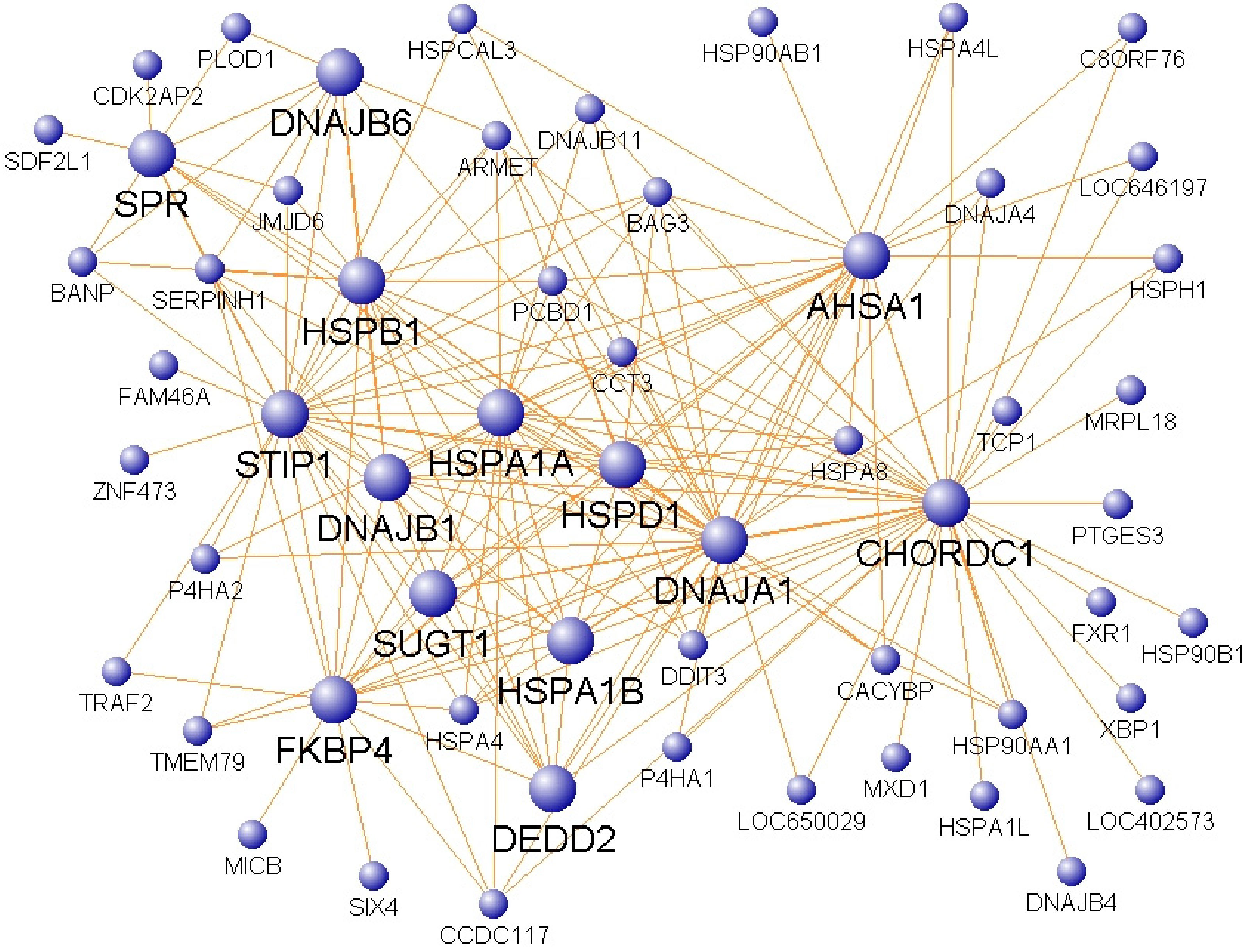

Top Genes
CHORDC1
HSPD1
DNAJA1
DNAJB6
AHSA1
HSPA1A
FXR1
HSPB1
HSPA1B
STIP1
PAPOLA
SUGT1
LOC402573
LOC63920
XBP1
IRF2BP2
PDIA4
SPR
MXD1
ARMET
HSPA1L
FKBP4
P4HA1
BAG3
CCDC117

Figure S8L (pink)

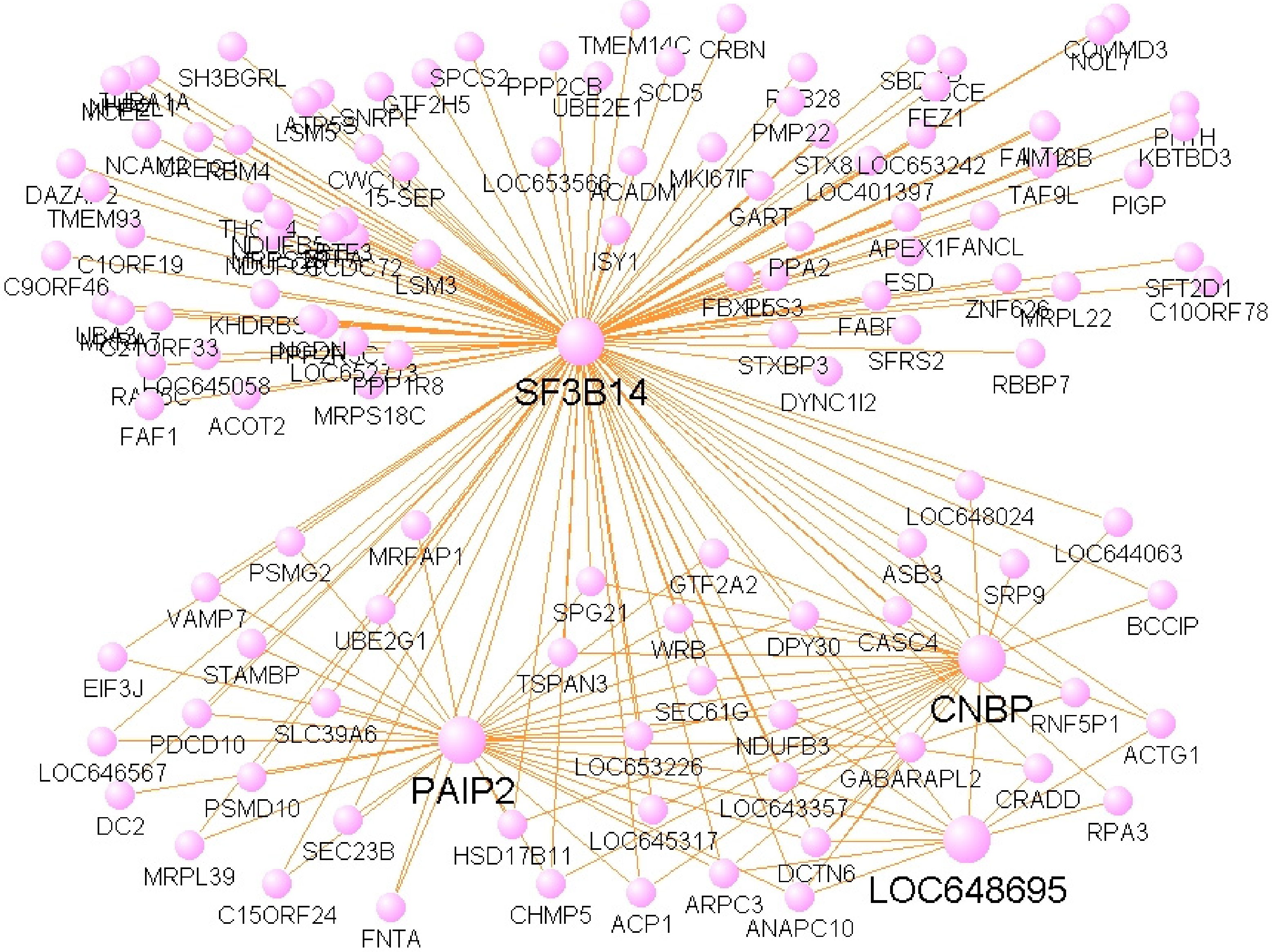

Top Genes
LOC401397
SF3B14
ESD
CNBP
PAIP2
GABARAPL2
CASC4
MRPS28
LOC648695
SEC23B
FAIM
GTF2H5
PIGP
LOC653226
GPM6B
STX8
UBE2A
PXMP3
PSMG2
PPP1R8
DAZAP2
TSPAN3
LOC647346
WRB
C9orf46

Figure S8M (purple)

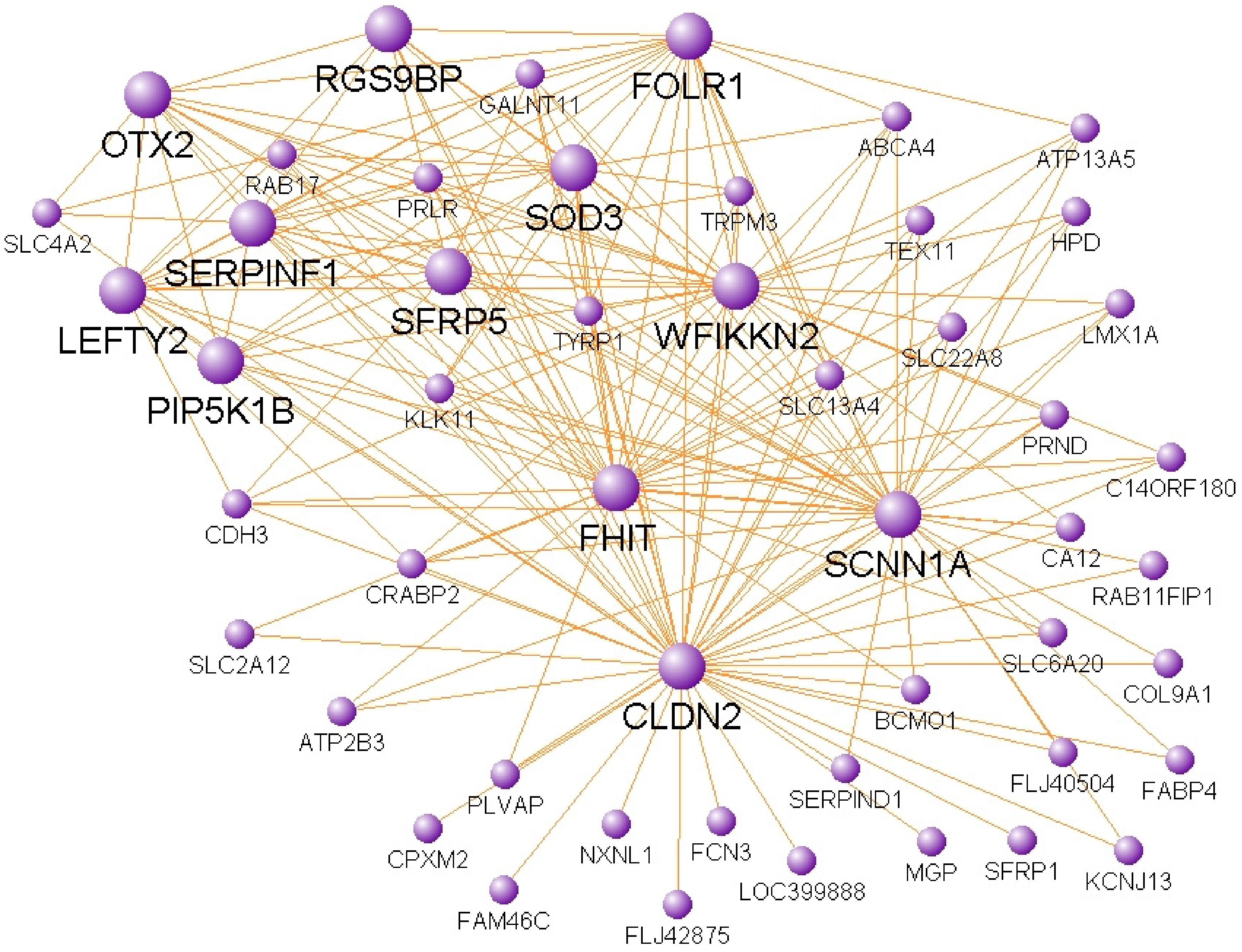

Top Genes
SLC4A2
CPXM2
PROS1
ROR2
WFIKKN2
FOLR1
SOD3
SLC16A10
RBBP8
LEFTY2
NXNL1
CLDN2
SLC6A20
C14orf180
CLIC6
C1QTNF5
FHIT
SCNN1A
SFRP1
SYTL3
SFRP5
NWD1
HPD
TGFBI
FAM46C

Figure S8N (red)

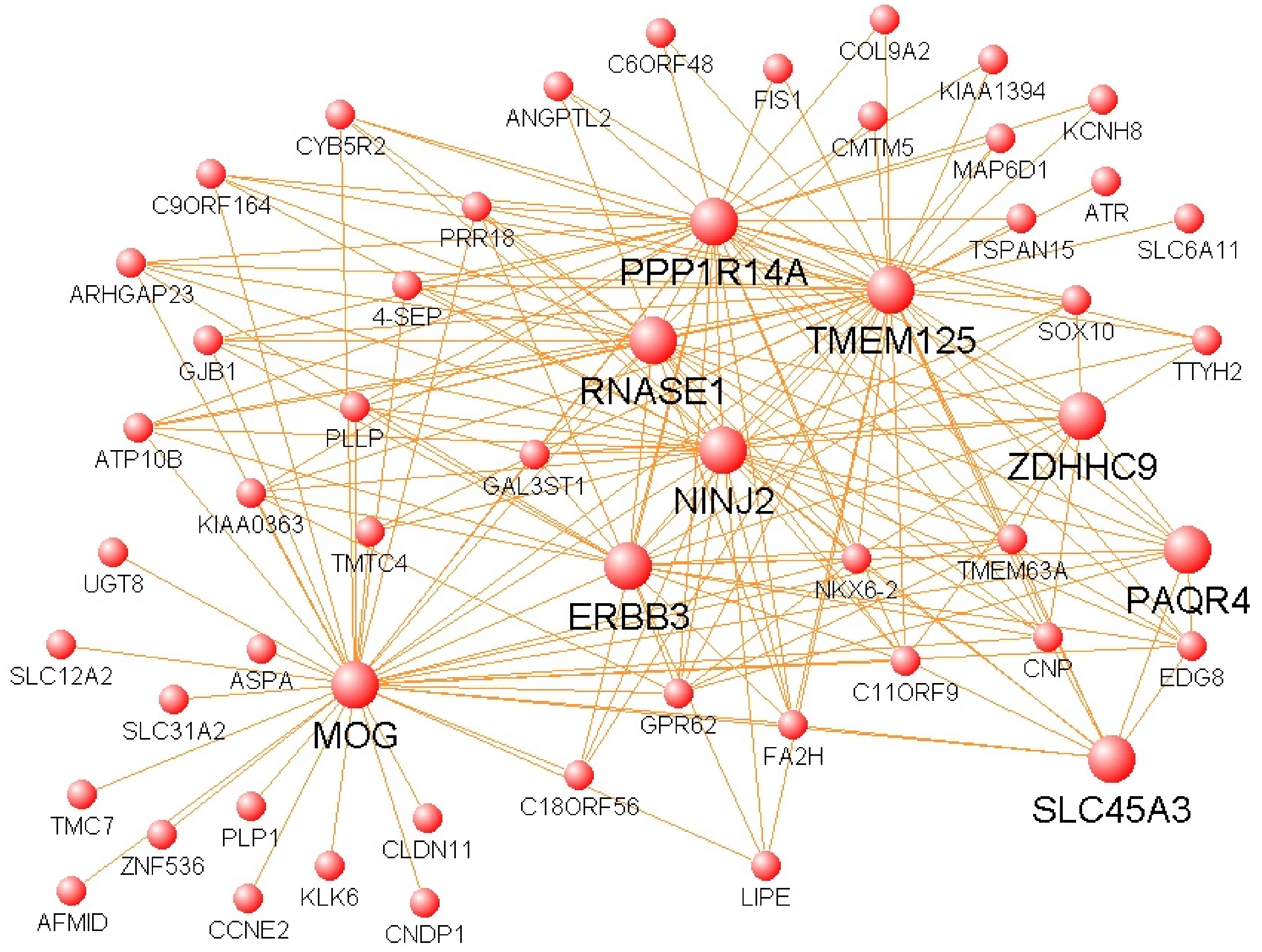

Top Genes

MOG
NINJ2
ICHTHYIN
HRASLS3
RNF13
FNTA
C5orf4
SLC45A3
RNASE1
MAL
ENDOD1
MAG
TMTC2
CLDN11
DNAH17
RNH1
ZNF536
LITAF
CMTM5
TMEM144
ATG4C
PLP1
RFFL
CD9
SLC35D2

Figure S8O (salmon)

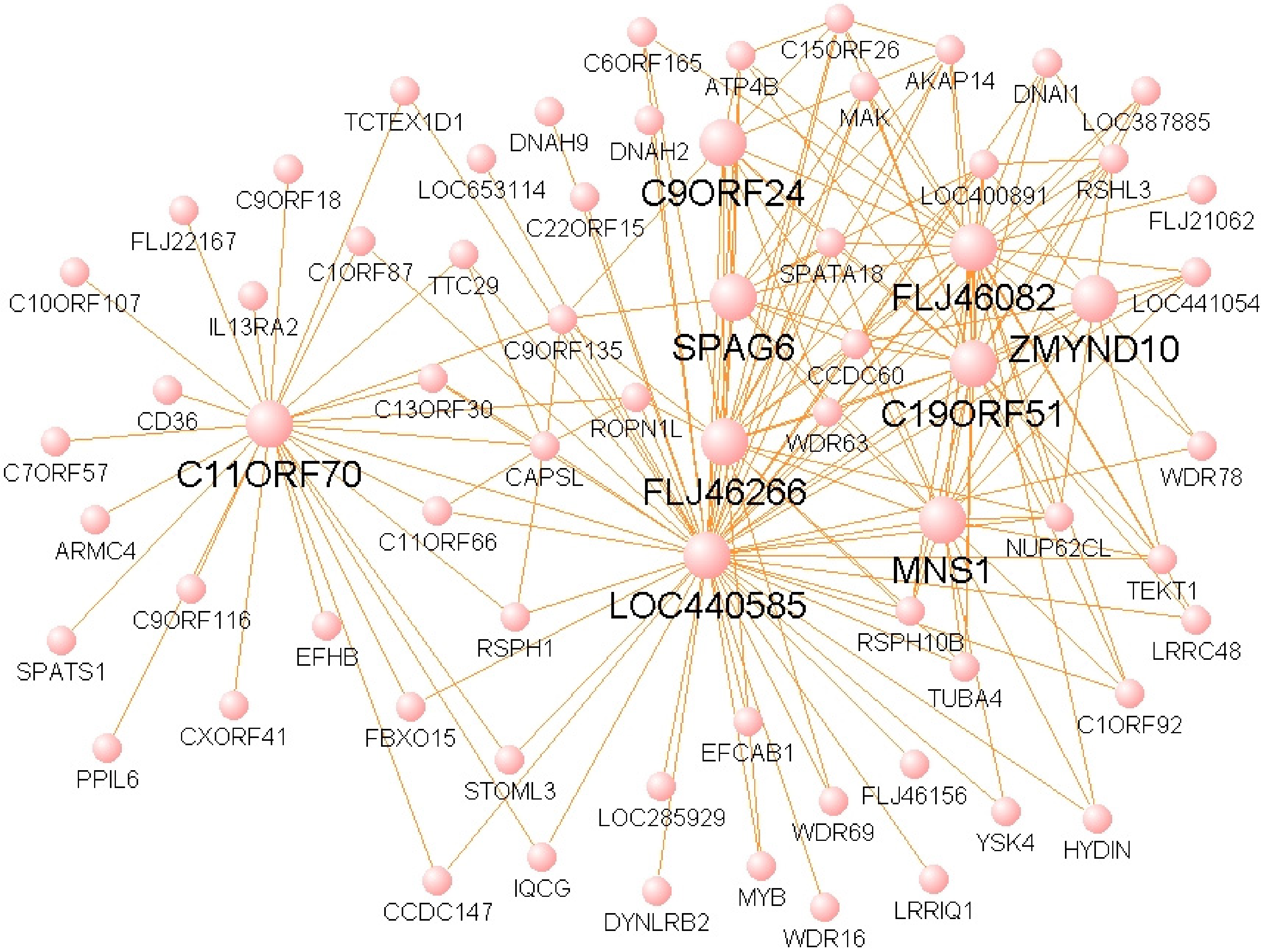

Top Genes
DYNLRB2
MNS1
LOC440585
ZMYND10
C9orf24
FLJ46082
C19orf51
LRRIQ1
CAPSL
TEKT1
C11orf70
FLJ46266
CCDC81
SLC47A2
NUP62CL
PLTP
C6orf165
C15orf26
HYDIN
EFCAB1
TTC26
SPAG6
LRRC23
TCTEX1D1
C22orf15

Figure S8P (tan)

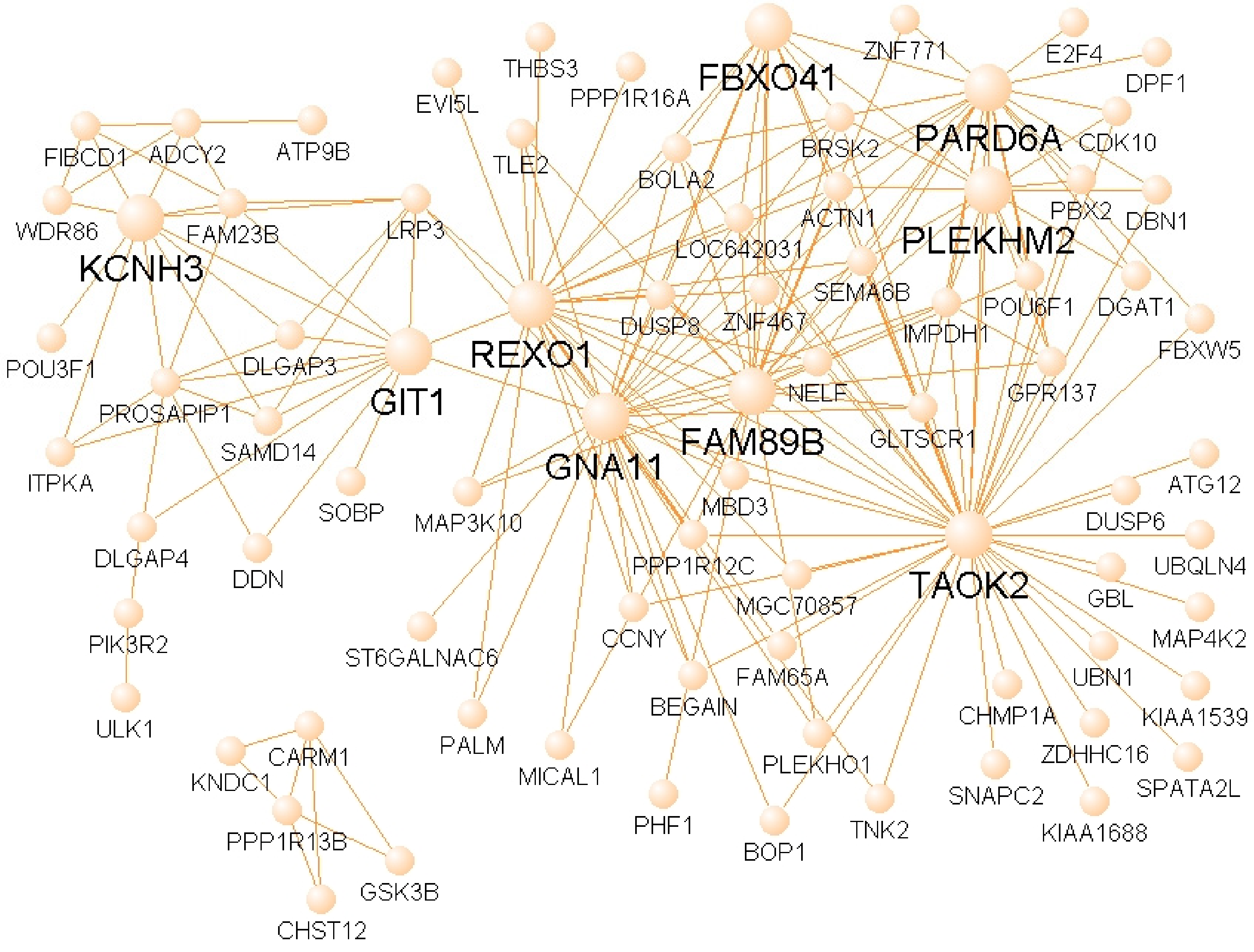

Top Genes
TAOK2
GNA11
RHOBTB2
BRSK1
PARD6A
ZER1
BOP1
ABHD8
KIAA1688
PLEKHM2
PPP1R13B
EGLN2
TNK2
REXO1
SAPS1
SPTBN2
CCM2
ZNF653
BAP1
SEMA6B
FAM89B
RIMS4
IMPDH1
CCNY
CENTA1

Figure S8Q (turquoise)

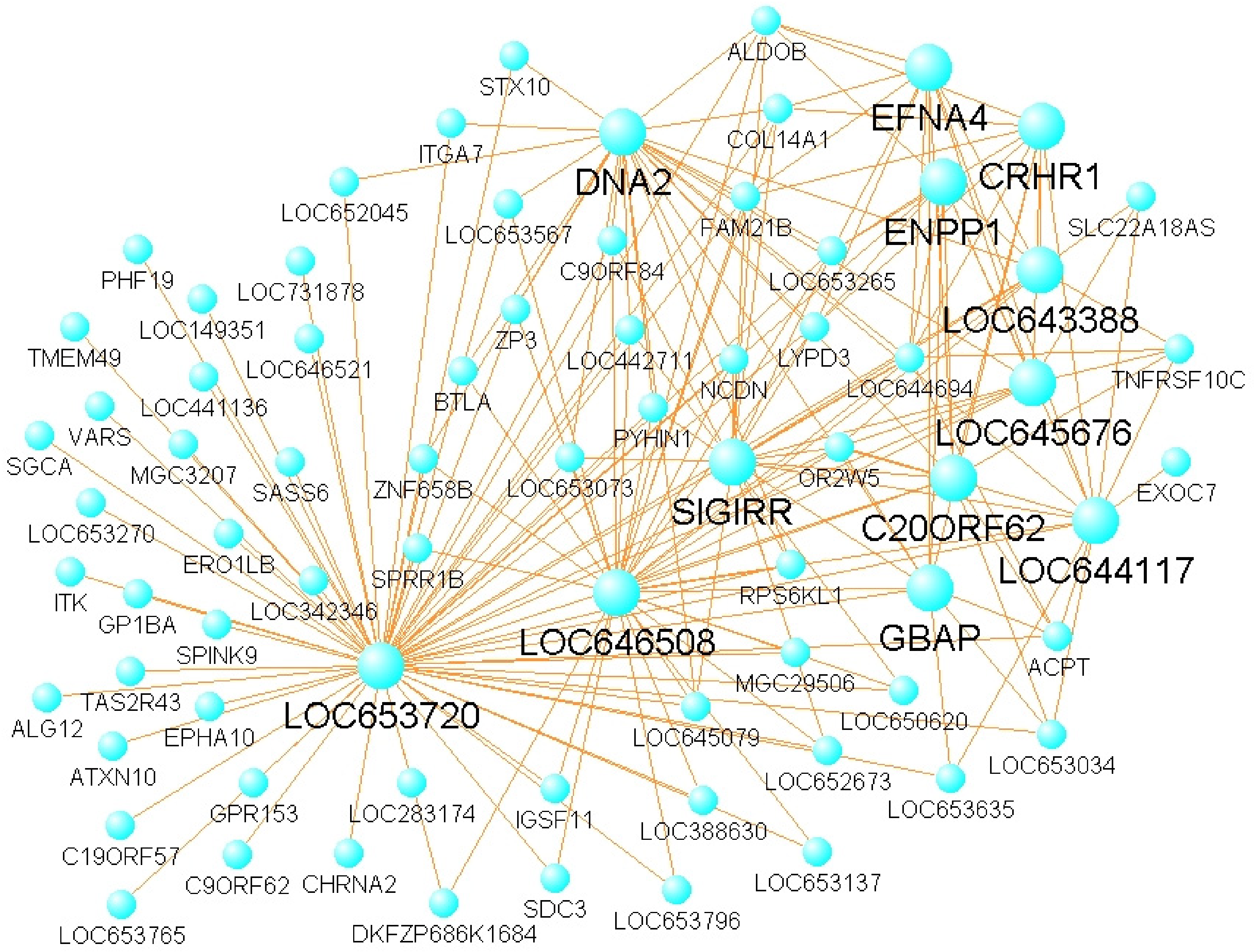

Top Genes
WNT5B
GDPD4
INSR
GPC6
ADPRH
TNFRSF10C
NRK
OR8G2
FCGR3B
CMTM3
BAZ2A
CHST2
AGTR2
FBXO3
ATPBD3
SLC22A18AS
LGI2
CCDC69
HOXD3
SSX5
CRHR1
MAGEA6
DPYSL5
SLC22A1
GAMT

Figure S8R (yellow)

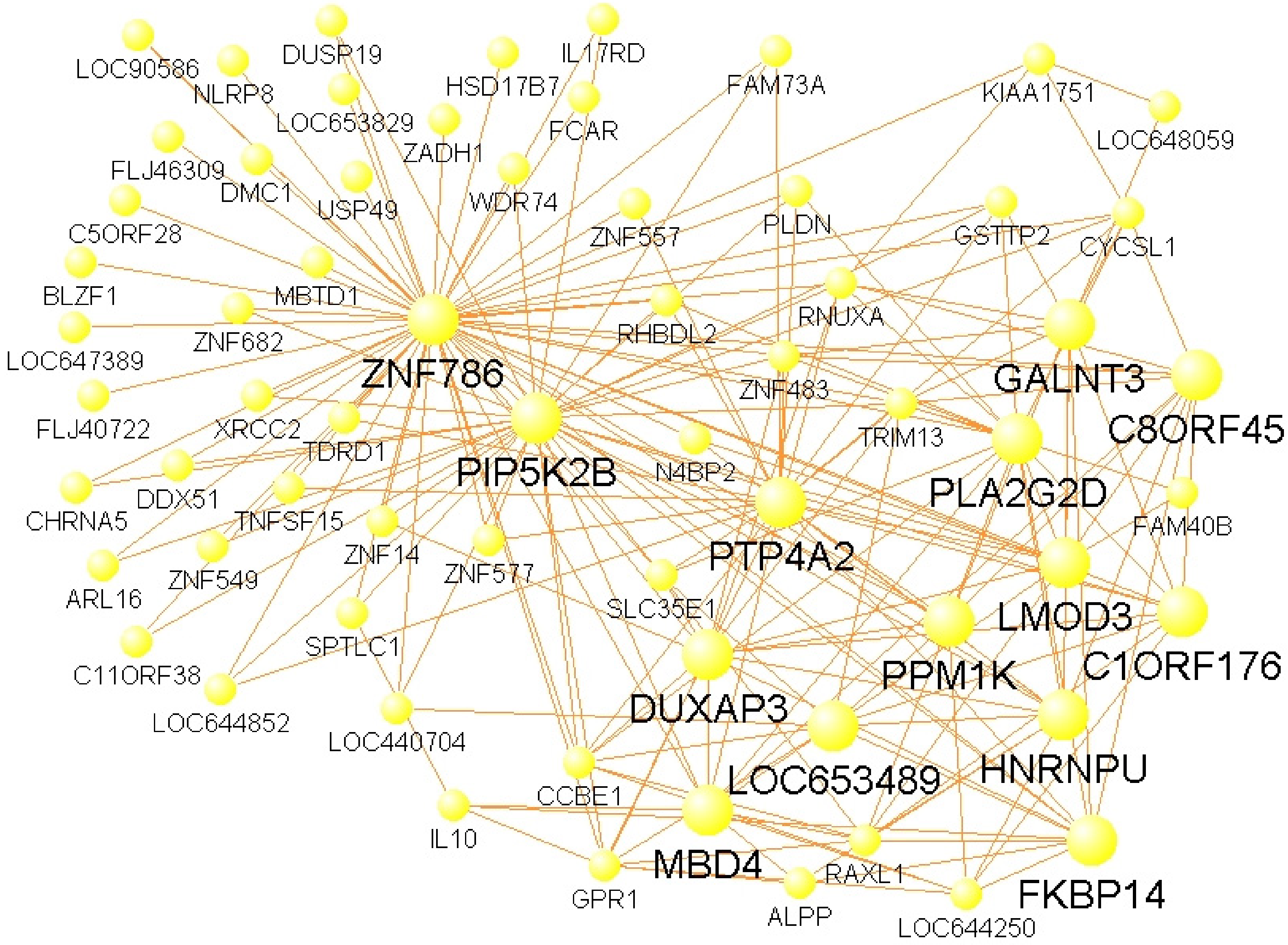

Top Genes
ZNF786
SLC4A5
C9orf130
RAXL1
LOC644250
ZNF223
ZNF652
CEP27
IL10
LOC653489
SDHALP1
ANKRD30B
PIP5K2B
FAM40B
TNFSF14
ALPP
PTPLAD2
DCLRE1C
PTP4A2
SPN
CSF2RA
ICA1
NUBPL
LOC653086
DENR

Supplementary Table 6

Gene	Module	Mean CT expression	Fold Change Br=2/Br=1	P-value Br=2/Br=1	Mean pyramidal expression	Fold Change NFT/noNFT	P-value NFT/noNFT
SIGLEC8	lightgreen	244.1	1.23	0.00371	48.7	1.90	0.00536
**TYROBP**	lightgreen	1716.4	1.53	0.00695	259.5	1.81	0.00110
MAFB	black	547.4	1.25	0.00872	321.0	1.73	0.00034
RNASET2	lightgreen	6657.1	1.35	0.00560	794.7	1.51	0.00898
TBXAS1	lightgreen	459.4	1.41	0.00210	61.7	1.43	0.00944
**CD74**	lightgreen	437.2	1.56	0.00993	666.5	1.72	0.00221
LOC653879	lightgreen	321.8	1.37	0.01028	771.6	2.03	0.00721
RNASE6	lightgreen	276.0	1.24	0.01303	41.8	2.17	0.00439
GPX1	lightgreen	4072.4	1.21	0.01463	1121.2	1.49	0.00135
**ITGAM**	lightgreen	346.4	1.36	0.00139	127.9	1.60	0.01550
DOCK2	lightgreen	439.4	1.33	0.00737	39.9	2.62	0.01790
GNLY	grey60	249.1	1.37	0.00333	40.4	4.69	0.02010
APOC2	lightgreen	441.1	1.30	0.01891	204.8	1.35	0.02010
SLC1A5	lightgreen	230.6	1.17	0.01978	148.2	2.45	0.02020
METTL7B	magenta	836.8	1.63	0.02230	234.0	1.41	0.02270
LST1	lightgreen	352.8	1.29	0.02402	70.0	2.73	0.00589
PYCARD	lightgreen	582.6	1.33	0.02496	79.3	2.09	0.00003
RASL10B	cyan	316.3	1.23	0.01859	217.8	1.21	0.02560
CTSS	lightgreen	205.5	1.23	0.00033	276.9	1.73	0.02610
HLA-DRB4	lightgreen	651.2	1.75	0.00006	96.7	1.77	0.02780
MYO1F	lightgreen	195.4	1.18	0.02833	142.8	1.69	0.00322
CPVL	purple	543.7	1.63	0.01193	66.2	1.42	0.02870
**RGS10**	lightgreen	1190.4	1.34	0.02878	93.2	1.77	0.02700
**HLA-DPA1**	lightgreen	1909.4	1.47	0.02937	1335.5	2.11	0.02820
ALOX5	lightgreen	606.2	1.31	0.02944	190.3	1.99	0.00122

Supplementary Table 6: Top NFT-related genes are mostly microglial. The top NFT genes in this table are each significantly upregulated in Braak=2 controls vs. Braak=1 controls (in this study; columns 3-5) and significantly increased in pyramidal cells containing NFTs relative to pyramidal cells not containing NFTs in entorhinal cortex of individuals with moderate AD (GSE4757; columns 6-8), both with $p<0.03$. Of the 25 genes meeting these requirements, 20 are in the light green module (column 2), and 5 are hubs in this module (in bold; see also Figure S6I).

Supplementary Table 7

Gene	Forward Primer	Reverse Primer	Purpose
GAPDH	GAAGGTGAAGGTCGGAGTCA	GATCTCGCTCCTGGAAGATG	Control gene
SPARCL1	GGTACCACTGAGCCTGGAGA	AAGGGGTTTTGTTGGAGGAC	CA1-specific in controls
NRIP3	TACCCCGGCATCTCAAAGTA	CTGTCTTCCCCATGATCAGC	CA3-specific in controls
ABHD12	TGCCATTCCATTTCTCATCA	ACATCTGGTCTTTGCCTTGG	CA3-specific in controls
TSPAN18	GATCATCTTCCTGGCAGAGC	TAAAGTCTTCAGGCCCGTTG	CA3-specific in controls
CXCL14	CGTGAAGAAGCTGGAAATGA	GGCGTTGTACCACTTGATGA	Down with AD in CA1
SEC14L5	TACCCGTTTGAGCTGGTCAT	AGCGTCCTCTCCTTCCAGTT	Down with AD in CA1
S100A6	ACACCCTGAGCAAGAAGGAG	CCCTTGAGGGCTTCATTGTA	Up with AD in CA1
GEM	CTTCGAGAAGGCATCTGAGC	CCCTCAAACAGCTCCTTCAC	Up with AD in CA1
HLA-DRB4	GTGACATTGACGGTGCTGAG	AGGAGGTCCTTCTGGCTGTT	Up with Braak stage in controls
ECHDC3	CGTGATTACCATGCCGAAGT	CAGGAGTGGCAAAAGAGGAC	Up with Braak stage in controls
PDPR	GGTCCTCTGCAAGAAACAGC	TCTGCTCAATGGTCAAGTGC	Up with Braak stage in controls
PDPR	AGGATGCAGTGGTGTCTTCC	TACTAACCGGCTCCTCGTTG	Up with Braak stage in controls
FGL2	TTTGGATGGCAAATGTTCAA	CCATGGTCTCCATGTCACAG	Up with Braak stage in controls
FCER1G	GGAGAGCCTCAGCTCTGCTA	TGGTGGTTTCTCATGCTTCA	Up with Braak stage in controls

Supplementary Table 7: Primers pairs used for qRT-PCR validations.
